# Electronic modulation of metal-support interactions improves polypropylene hydrogenolysis over ruthenium catalysts

**DOI:** 10.1038/s41467-022-32934-5

**Published:** 2022-09-03

**Authors:** Pavel A. Kots, Tianjun Xie, Brandon C. Vance, Caitlin M. Quinn, Matheus Dorneles de Mello, J. Anibal Boscoboinik, Cong Wang, Pawan Kumar, Eric A. Stach, Nebojsa S. Marinkovic, Lu Ma, Steven N. Ehrlich, Dionisios G. Vlachos

**Affiliations:** 1 Center for Plastics Innovation, University of Delaware, 221 Academy St., Newark, DE 19716 USA; 2 Department of Chemical and Biomolecular Engineering, University of Delaware, 150 Academy St., Newark, DE 19716 USA; 3 Department of Chemistry and Biochemistry, University of Delaware, Newark, DE 19716 USA; 4 Center for Functional Nanomaterials, Brookhaven National Laboratory, 735 Brookhaven Ave, Upton, NY 11973 USA; 5 Department of Materials Science and Engineering, University of Pennsylvania, Philadelphia, PA 19104 USA; 6 Department of Chemical Engineering, Columbia University, 500W 120th St., New York, NY 10027 USA; 7 National Synchrotron Light Source II, Brookhaven National Laboratory, Upton, NY 11973 USA

**Keywords:** Heterogeneous catalysis, Porous materials, Catalytic mechanisms

## Abstract

Ruthenium (Ru) is the one of the most promising catalysts for polyolefin hydrogenolysis. Its performance varies widely with the support, but the reasons remain unknown. Here, we introduce a simple synthetic strategy (using ammonia as a modulator) to tune metal-support interactions and apply it to Ru deposited on titania (TiO_2_). We demonstrate that combining deuterium nuclear magnetic resonance spectroscopy with temperature variation and density functional theory can reveal the complex nature, binding strength, and H amount. H_2_ activation occurs heterolytically, leading to a hydride on Ru, an H^+^ on the nearest oxygen, and a partially positively charged Ru. This leads to partial reduction of TiO_2_ and high coverages of H for spillover, showcasing a threefold increase in hydrogenolysis rates. This result points to the key role of the surface hydrogen coverage in improving hydrogenolysis catalyst performance.

## Abstract (web summary)

Catalytic pathways of plastic waste valorization to lubricants are attractive avenues to foster circular economy. Tuning of catalyst electronic properties allows to significantly improve its activity due to boosted hydrogen storage on the surface.

## Introduction

Plastic waste represents a significant threat to the environment due to its leakage into the oceans and soil. Plastic consumption increased drastically during the recent pandemic^1^, triggering a soaring expansion of incineration, causing extensive CO_2_ emissions^2^, and landfilling of consumer packaging materials, foams, films, and personal protection equipment^3^. Polypropylene (PP) is a large-volume polymer frequently used in packaging, fabrics, and textiles, including face masks. Mechanical recycling fails to deal with mixed PP waste streams since composites usually contain pigments, dyes, antioxidants, and plasticizers, leading to an inferior product. PureCycle^4^ has demonstrated that solvent extraction can provide consumer-grade PP, but solvent use increases cost and complexity. A possible approach to extend the life cycle is the catalytic conversion of PP waste under solvent-free conditions.

Hydrogenolysis is a low-energy valorization route of PP and polyethylene (PE)^5^, producing liquid products, including lubricant base oil^6^. Ru and Pt nanoparticles on carbon or oxide supports have been the hydrogenolysis catalysts of choice^7–11^. Ru, in particular, is more active than Pt but forms copious amounts of methane, and reaction times are long. Its performance varies widely with the support, but the reasons remain unclear^7,10–14^. For example, over Ru/ZrO_2_, the strong PE binding leads to the over-cracking of the reaction intermediates to methane^15^. On Ru/WO_x_/ZrO_2_, on the other hand, hydrogen spillover from Ru stores hydrogen in reducible surface polytungstate domains, thus effectively removing reaction intermediates and suppressing methane formation. Recent work on Ru deposited on redox-active CeO_2_ support showed 83% liquid yield. Controlling hydrogen availability on the catalyst and polyolefin binding may effectively control the chemistry, but ways to achieve this are lacking.

Here, we introduce a systematic approach to tune the reducibility of the TiO_2_ support and the metal-support interactions (MSI). Formation of TiO_x_ overlayer reported initially in ref. 16 is known to poison the metal surface and inhibit hydrogen chemisorption. For the Ru/TiO_2_ catalyst, the MSI enhanced by the lattice matching between RuO_x_ and rutile TiO_2_ leads to higher catalytic activity in CO_2_ hydrogenation^17^. We perform extensive characterization and correlate the electronic properties and hydrogen storage to catalytic performance in PP hydrogenolysis. We show that extensive reduction of TiO_2_ via hydrogen spillover from Ru modifies the hydrogen storage capacity of the Ru nanoparticles. This boosts the hydrogenolysis activity and reduces the liquid molecular weight and the reaction time.

## Results

### Ru/TiO_2_ catalyst characterization

**Fig. 1:**
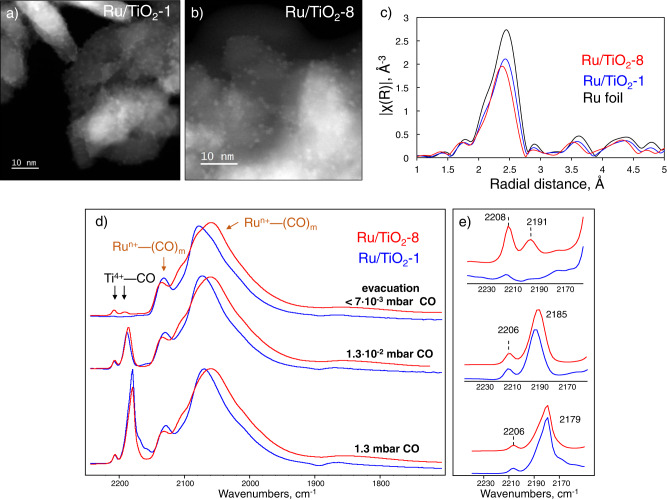
Catalyst characterization. **a**, **b** STEM images of Ru/TiO_2_-1 and Ru/TiO_2_-8, respectively; **c** EXAFS spectra of two samples and Ru foil standard; **d** FTIR of adsorbed CO at −296 °C at different CO pressures; **e** Zoom in of the 2240–2150 cm^−1^ region.

We changed the pH in the Ru deposition step, using aqueous NH_3_, to modify the catalyst (catalysts are labeled as Ru/TiO_2_-x, where x indicates the synthesis pH) while ensuring similar Ru loadings (~3 wt%), surface areas (100 m^2^/g) (Table 1), and pore volume. According to XPS quantitative analysis, all samples have similar surface Ru/Ti atomic ratios consistent with the same Ru loadings. XRD patterns (Supplementary Fig. 1) show relatively broad reflections of the TiO_2_ anatase support with no sign of crystalline Ru. Ex situ UV-Vis spectra reveal comparable bandgap energies, typical of pure anatase TiO_2_ (Table 1). All samples contain predominantly 1.4–1.6 nm Ru nanoparticles evenly distributed on TiO_2_ (STEM images in Fig. 1a, b and particle size distribution in Supplementary Fig. 2). TiO_2_ consists of elongated cylindrical, highly crystalline particles of 35–40 nm length and ~10 nm diameter. In situ Ru K-edge XANES spectra (Supplementary Fig. 3) after pre-reduction with H_2_ at 250 °C indicate metallic Ru^0^ and EXAFS spectral analysis (Table 1, Fig. 1c, Supplementary Fig. 3, Supplementary Table 1) show a coordination number of ~8.5, consistent with the STEM images.

**Table 1:** Ru/TiO_2_ catalyst characterization

Catalyst property	Ru-TiO_2_-1	Ru-TiO_2_-8	Ru-TiO_2_-12
Ru loading, wt%	3.1	3.1	3.3
(Ru/Ti)_bulk_^a^	0.025	0.025	0.027
(Ru/Ti)_surf_^b^	0.19	0.20	0.22
d(Ru)_TEM_, nm	1.4	1.2	1.4
H_chem_, μmol/g^c^	270	180	140
D_H_, %^d^	88	59	43
E_g_, eV^e^	3.2	3.2	2.8
S_BET_, m^2^/g	100	90	95
V_p_, cm^3^/g	0.35	0.33	0.33

^a^Measured by XRF.

^b^Measured by XPS.

^c^Chemisorbed hydrogen measured by pulse chemisorption.

^d^Apparent dispersion estimated from pulse chemisorption assuming H_chem_/Ru_surf_ ratio 1.

^e^Optical bandgap measured by UV-Vis spectroscopy.

TGA results (Supplementary Fig. 4) show that ~10 mmol//${{{{{{\rm{g}}}}}}}_{{{{{{{\rm{TiO}}}}}}}_{2}}$ of NH_3_ is retained after impregnation, an order of magnitude higher than Ti^4+^ on the surface^18^. NH_3_ desorbs in H_2_/He at ~250 °C, indicating strong interaction with the catalyst. NH_3_ can reduce and modify the TiO_2_ surface after high-temperature treatment^19,20^. Based on DRIFTS spectra (Supplementary Fig. 5) of Ru/TiO_2_-8 directly after impregnation and drying and before the reduction in H_2_, ammonia is adsorbed in several modes. Two possible mechanisms are proposed to explain the NH_3_ role during sample pre-reduction (Supplementary Figs. 6–8). XPS analysis shows no signs of N-doping of TiO_2_ after reduction.

To investigate the modification of the Lewis acidity, we used FTIR of adsorbed CO at −196 °С (Fig. 1d, e). At high CO pressure, Lewis acid site bands appear at 2206 and 2179 cm^−1^ due to highly electrophilic four-coordinated Ti^4+^(O_4_) sites on the particle edges and the less acidic five-coordinated Ti^4+^(O_5_) sites on the 101 crystal planes, respectively^21^. The concentration of Ti^4+^(O_4_) sites is similar in both samples. The intensity of both bands decreases with evacuation time, but the 2206 cm^−1^ band is reduced more slowly. Ru/TiO_2_-8 retains some CO on both sites even after prolonged evacuation, whereas Ru/TiO_2_-1 only marginally on the stronger Ti^4+^(O_4_) sites (Fig. 1e), indicating stronger binding and Lewis acidity on the former (Supplementary Fig. 9). DRIFTS demonstrates a smaller density of surface Ti-OH groups on the Ru/TiO_2_-8 and Ru/TiO_2_-12 than on Ru/TiO_2_-1 (Supplementary Fig. 10). Partial dehydroxylation enhances the Lewis acidity of Ti^4+^ sites due to the redistribution of the electron density on the surface^22^.

In addition to Ti^4+^–CO adducts, FTIR spectra show that Ru particles get partially oxidized by CO forming multiple Ru^n+^(CO)_m_ (m = 1–4) carbonyls with ν(CO) vibration at 2136, 2106, 2084, and 2055 cm^−1^ (Supplementary Fig. 11)^23^. Due to the low-temperature CO dissociation, clusters of Ru^n+^ and Ru^0^ species form. The stronger interaction of TiO_2_ and Ru and the TiO_x_ peripheral layer shifts the broad ν(CO) band to lower wavenumbers^24^ (from 2077 to 2059 cm^−1^, Fig. 1d), epitomizing a proximal MSI in the Ru/TiO_2_-8 catalyst.

### Hydrogen binding on Ru/TiO_2_

H_2_ temperature-programmed desorption (TPD) (Supplementary Fig. 12) indicates a similar amount of strongly chemisorbed hydrogen on the Ru (low-temperature peak at 160 °C)^25^ and TiO_2_ (high-temperature shoulder at 250–300 °C)^26^. Temperature-programmed reduction (TPR) in H_2_ flow shows a spike in hydrogen adsorption at 80–90 °C, due to binding on Ru, and a broad peak starting at ca. 250 °C, due to partial TiO_2_ reduction (Supplementary Fig. 13). Interestingly, Ru/TiO_2_-8 and Ru/TiO_2_-12 show more pronounced hydrogen uptakes at 150-200 °C than Ru/TiO_2_-1. Hydrogen pulse chemisorption at 35 °C (Table 1) shows that Ru/TiO_2_ samples have different hydrogen uptakes. During an experiment, hydrogen can spillover to the TiO_2_ support obscuring the real Ru dispersion values. Thus the total H_2_ uptake inferred from pulse chemisorption depends on the spillover capacity of TiO_2_, rendering accurate quantification of the exposed Ru surface area impossible with the available methods. Also, the pretreatment temperature before pulse chemisorption (300 °C) is insufficient to remove all chemisorbed hydrogen from the sample. Thus, apparent uptakes may be higher for catalysts with weaker chemisorption.

**Fig. 2:**
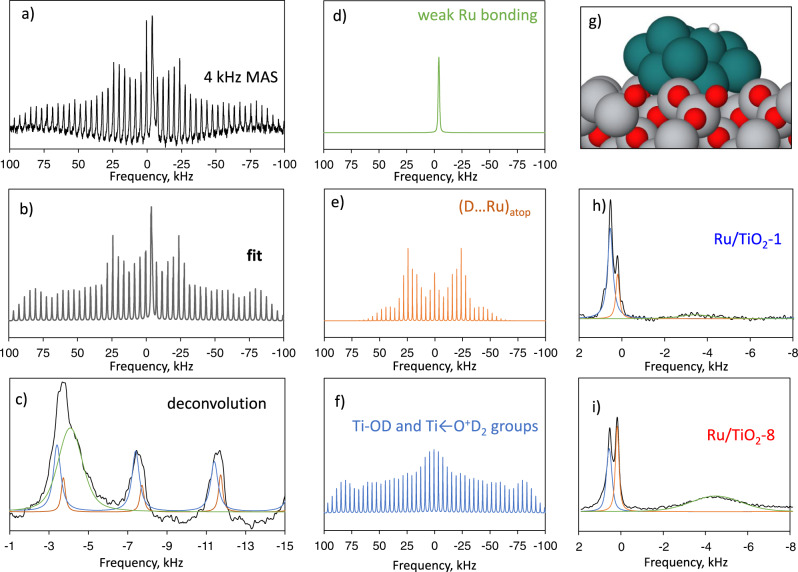
Hydrogen chemisorption on Ru/TiO_2_. **a** Solid-state ^2^H MAS NMR spectra of Ru/TiO_2_-8 measured at 4 kHz of spinning. **b** Fitting of experimental spectra. **c** Zoom in low-frequencies alongside three components used for fitting, resolved via deconvolution. **d**–**f** Deconvoluted signals of weakly bonded D on Ru (**d**), strongly bonded D on Ru in atop configuration, **e**, and Ti-OD and Ti←O^+^D_2_ groups on TiO_2_ (**f**). **g** DFT-deduced structure of atop bonded D on a Ru cluster. **h**, **i** Spectra at 10 kHz MAS for Ru/TiO_2_−1 (**h**) and Ru/TiO_2_−8 (**i**).

To decouple Ru from support contributions to the hydrogen binding and activation, we used ^2^H MAS NMR of chemisorbed D_2_ (Fig. 2). Chemisorption of ^2^H on Ru clusters is accompanied by quadrupole interactions with the surrounding electric field gradient (EFG), leading to characteristic sidebands in the NMR spectra. Spectra contours depend on the quadrupole coupling constant (Q_cc_), a function of the largest EFG component (V_zz_), and the asymmetry parameter *η*, defined as (V_xx_ − V_yy_)/V_zz_. One can obtain the EFG parameters, sensitive to the deuteron local surroundings and the binding type^27,28^.

Figure 2a shows a complex line shape after sample reduction followed by saturation with 1 torr of D_2_. Deconvolution yields several components (Fig. 2b, c; high-resolution data at 10 kHz shown in Supplementary Fig. 14). The first component (Q_cc_~123–130 kHz/*η*~0.7–1.0) corresponds to Ti-OD formed by deuterium spillover, confirmed by measuring a D_2_O-treated pure TiO_2_ sample (Fig. 2f, Supplementary Fig. 15, Supplementary Table 2). Also, a simple H/D isotope exchange leads to the formation of Ti-OD groups^29^, and their presence in the spectra is not a fingerprint of spillover.

The latter peak has a symmetric EFG (*η*~0.1) and low Q_cc_ of ca. 70 kHz, distinctly different from the Ti-OD signal (Fig. 2e, Supplementary Table 3), and consistent with Ru_n_-D hydrides^27^. The second component corresponds to deuterons bonded to Ru in atop conformation (see parameters in Supplementary Tables 3 and 4), confirmed using DFT calculations (Supplementary Fig. 16). Figure 2g shows an optimized structure of a Ru cluster on TiO_2_ with a D atom, in good agreement with a previous report^30^, underscoring the preferential formation of atop H instead of (Ru)_2_-H bridging binding. The EFG parameters depend on the Ru particle size and the support. For an unsupported Ru_12_ cluster, EFG is more symmetric, unlike the experimental data. Smaller or larger clusters than Ru_12_ on TiO_2_ provide a less adequate EFG. Thus, ^2^H NMR, combined with DFT, provides insights into MSI and particle size effects.

The third component gives a very broad single line with no sidebands (Fig. 2d) at a low resonance frequency (−4 to −4.4 kHz, or −53 to −57.3 ppm). These features are not standard for Ru_n_-D hydrides, observed previously for free-standing Ru clusters^27^. The negative chemical shift is similar to chemisorbed hydrogen on Ru particles on SiO_2_ and TiO_2_, inferred from static ^1^H NMR^31,32^. The deuteron interaction with the Ru conduction electrons leads to the so-called Knight shift responsible for the −53 ppm peak^33^. On Ru/SiO_2_, this peak stems from the overlap of different signals due to strongly and weakly chemisorbed hydrogen^31^. We exclude deuteron binding to an oxygen vacancy in TiO_2_ because it would lead to resonances close to 0 kHz at −1.07 ppm^34^ or −0.66 ppm and −0.78 ppm^35^. Static ^1^H NMR studies showed that the exact position and linewidth are highly affected by Na doping of a Ru/TiO_2_ catalyst^36^, i.e., this deuteron is sensitive to interactions with the support. In our case, direct interaction via Fermi contact between Ti^3+^ paramagnetic sites on the partially reduced TiO_x_ support can affect the position and intensity of this peak.

Sample evacuation at 100 °C leads to a significant reduction in the intensity of the −53.2 ppm peak and shifting to −45.8 ppm (lower Knight shift) (Supplementary Figs. 17, 18, Supplementary Table 5). Interestingly, the ratio of Ti-OD and Ru-D_atop_ groups remains constant, indicating comparable stability. Thus, the resonance at −53.2 ppm corresponds to the weakly bonded deuteron, not entirely captured in the TPD. Unlike the Ru-D_atop_ signal, these deuterons are trapped by the Ru’s conduction electrons or some Ti^3+^ charged center of the support. Based on the thermal stability of the adsorbed deuterium studied by ^2^H MAS NMR and TPD, all three types of surface deuterons are stable at 25 °C and desorb only upon heating.

The ^2^H MAS NMR spectra reveal significant differences in the deuterons (Fig. 2h,i, Supplementary Fig. 19). The NH_3_-treated samples have a much higher content of Ru-bonded deuterium than Ti-OD groups. Higher resolution 10 kHz MAS spectra (Fig. 2h, i) show that Ru-related peaks at 0.18 kHz (2.1 ppm) and ~−4.49 kHz (−58.0 ppm) over Ru/TiO_2_-8 are more pronounced than on Ru/TiO_2_-1 The data combined (Supplementary Table 6, 7) reveals that Ru/TiO_2_-8 has a higher absolute amount of Ru-bonded deuterium. This was further supported by a direct comparison of relative distributions of different deuterons. Thus, NH_3_-treated supports promote hydrogen binding to Ru clusters.

### Dynamics of hydrogen-Ru/TiO_2_ interactions

**Fig. 3:**
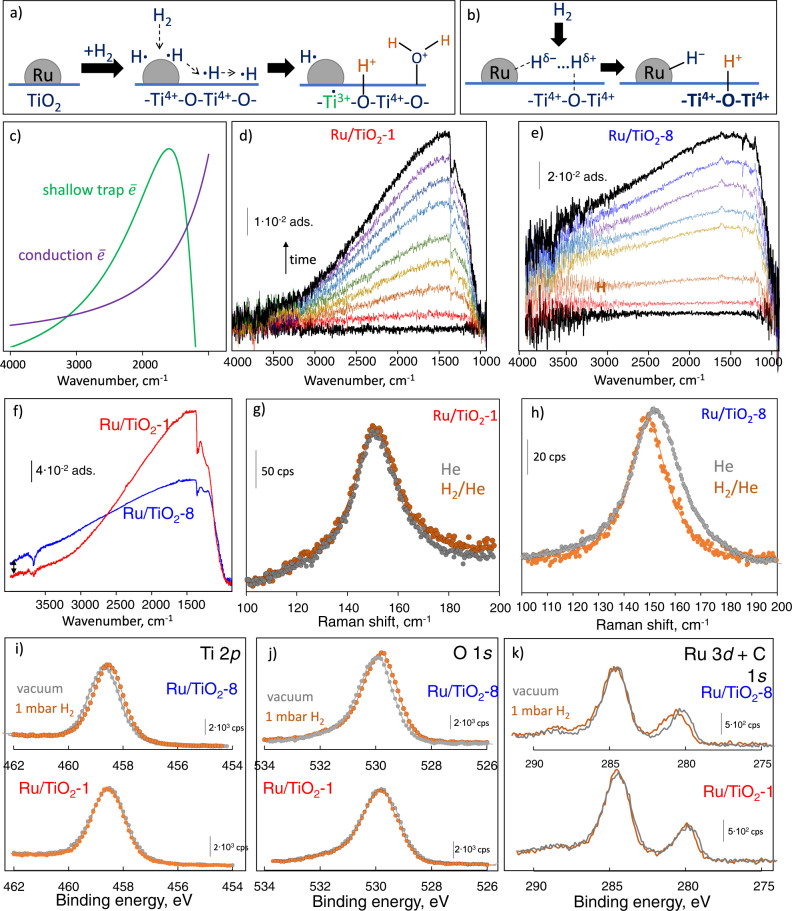
Hydrogen activation over Ru/TiO_2_. **a** Hydrogen spillover schematic over Ru/TiO_2_. **b** H_2_ binding on the metal-support interface. **c** Theoretical IR spectra for free electrons in the TiO_2_ conduction band and Ti^3+^ shallow trapped electrons. **d**, **e** Transmission FTIR transient spectra when switching from pure He to H_2_/He flow at 200 °C on Ru/TiO_2_−1 and Ru/TiO_2_−8, respectively. **f** Steady-state FTIR spectra for both samples at 250 °C and 0.14 bar H_2_. **g**, **h** In situ Raman spectra for Ru/TiO_2_-1 and Ru/TiO_2_-8 at 200 °C in He and H_2_/He flows, respectively (lines show Lorentzian curve fitting). **i**–**k** NAP-XPS of Ru/TiO_2_ samples under high vacuum and 1 mbar H_2_ pressure at 200 °C in T *2p* (**i**), O *1* *s* (**j**), and Ru *3d* (**k**) regions.

Hydrogen can partially reduce the TiO_2_ support by hydrogen spillover (Fig. 3)^29^, forming OH groups, and injecting electrons into TiO_2_, creating Ti^3+^ sites as a new state in the bandgap (Fig. 3a)^37^. Thermal excitation could also cause electron delocalization and populate the titania conduction band (CB)^38^. Specifically, hydrogen spillover creates a Ti^3+^ shallow trap, 0.1–0.2 eV below the CB edge, and a broadband in the FTIR spectra (Fig. 3c), frequently used to study spillover^39,40^. Electrons residing in the CB produce a power-law type spectrum distinct from shallow traps.

On Ru/TiO_2_-1, hydrogen binding leads primarily to shallow trap electrons (Fig. 3d). Due to the reversible spillover, this partial reduction is sensitive to temperature and hydrogen pressure (Supplementary Figs. 20, 21). A much broader background increase is seen on Ru/TiO_2_-8 due to the additional partial CB filling (Fig. 3e, Supplementary Fig. 22). The Ti-OH groups give a slight negative peak of ν(OH) at 3665 cm^−1^, and a new peak for δ(HOH) at 1614 cm^−1^ emerges due to the recombinative dehydroxylation of vicinal Ti-OH groups into water. Water formation stimulates the population of CB electrons^41^. FTIR shows that Ru/TiO_2_-1 is mainly reduced into localized Ti^3+^ states, whereas NH_3_-treated samples produce delocalized CB electrons (Fig. 3f). Hydrogen spillover is activated only at sufficiently high H coverage on Ru particles^30^, i.e., the low H coverage on the Ru/TiO_2_-1 sample (detected by ^2^H NMR) does not promote spillover and TiO_2_ reduction to the same extent.

In situ Raman spectroscopy at 200 °C in He and H_2_/He flows (Fig. 3g, h) corroborates this result. The *E*_g_(1) vibrational mode of TiO_2_ anatase (~144 cm^−1^) is sensitive to the concentration of electrons^42^. On Ru/TiO_2_-1, the peak position is unaffected by hydrogen (Fig. 3g). In contrast, on Ru/TiO_2_-8 it shifts by ~3 cm^−1^ due to forming CB electrons by hydrogen; shallow traps (Ti^3+^ states) do not contribute.

The extent of reduction and associated charge transfer were monitored using NAP-XPS. Upon introducing hydrogen at 200 °C to Ru/TiO_2_-8, the Ti 2*p*_3/2_ peak (458.69 eV; Fig. 3i, Supplementary Fig. 23, Supplementary Table 8) and the main lattice oxygen peak in the O 1 *s* region^43^ (Fig. 3j) shift to lower binding energies by 0.15 eV due to modest support reduction. No shifts are evident on Ru/TiO_2_-1, indicating low receptivity toward hydrogen. Since the penetration depth of NAP-XPS corresponds to 2–3 nm, the Ru 3*d* and Ti 2*p* signals are collected from the whole Ru nanoparticle volume and 1–2 atomic layers of the underlying TiO_2_ support^44^. Thus, changes in the XPS spectra reflect charge distribution and MSI, but are not sensitive to the formation of the narrow TiO_x_ peripheral layer observed in the FTIR of CO experiments (Fig. 1d).

The Ru *3d* doublet overlaps with the C 1 *s* signal caused by carbonaceous deposits on the initial TiO_2_ (Fig. 3k). Ru/TiO_2_-1 and Ru/TiO_2_-8 have slightly different binding energy (BE): 279.70 and 280.12 eV, respectively (Supplementary Table 8). Such values are typical for small Ru particles on TiO_2_^44^. Upon exposure of Ru/TiO_2_-1 to hydrogen at 200 °C, the BE of the Ru 3*d*_5/2_ peak increases by 0.12 eV; over Ru/TiO_2_-8, it shifts by 0.61 eV, indicative of Ru^δ+^ species. H_2_ activation at the Ru-TiO_2_ interface leads to negatively charged H^**−**^ attached to the metal and H^+^ binding to the nearest oxygen atom (Fig. 3b)^45^. This heterolytic hydrogen activation leads to a partial positive charge on Ru due to forming negatively charged hydrides (H^**−**^) (Fig. 3b). The direct charge transfer from Ru to TiO_2_ CB leads to a partial reduction of TiO_2_, as reported in CO_2_ reduction^46^. Ru^δ+^-H^**−**^ pairs upon H_2_ adsorption were also reported on Ru/carbon nanotubes^47^. Ru on NH_3_-treated supports allows spillover of H_2_, forming delocalized electrons, reducing the support more extensively, and increasing the positive charge of Ru.

### PP hydrogenolysis

**Fig. 4:**
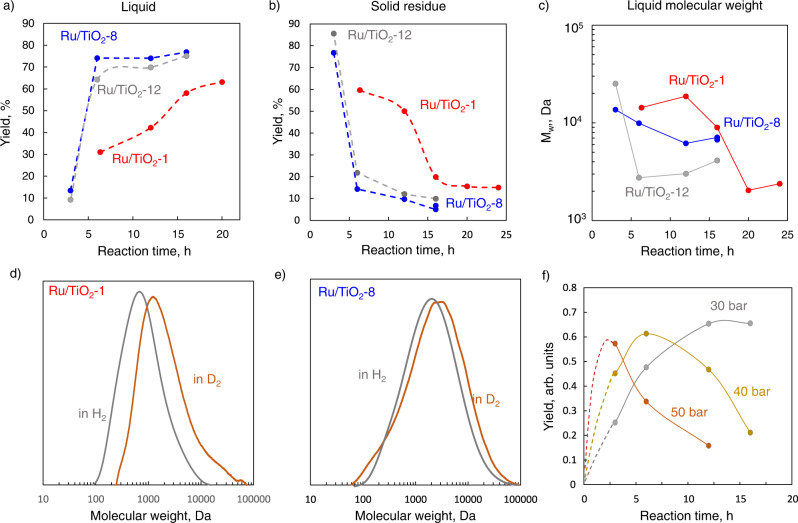
Ru/TiO_2_ hydrogenolysis performance in PP deconstruction. **a**, **b** Yields of liquids (**a**) and solid residue (**b**) vs. time. **c** M_w_ of liquid vs. time. **d**, **e** Effect of substitution of H_2_ with D_2_ on liquid molecular weight distribution over Ru/TiO_2_-1 (**d**) and Ru/TiO_2_-8 (**e**) catalysts. **f** Effect of hydrogen pressure on liquid yield over Ru/TiO_2_-1 (the dotted line extrapolates the data, using a simple A → B → C kinetic scheme). Conditions: 250 °C, 30 bar H_2_, 2 g PP, 0.05 g catalyst (**a**, **c**) or 0.1 g catalyst (**b**, **d**–**f**) for a PP/catalyst ratio of 40 and 20, respectively.

PP hydrogenolysis data over the three Ru/TiO_2_ samples with two polymer/catalyst weight ratios, 20 and 40, is shown in Fig. 4a–c and Supplementary Figs. 22, 23. The liquid yield increases with time and reaches 74% (Ru/TiO_2_-8) and 65–70% (Ru/TiO_2_-12) at 6 h, compared to only 63% at much longer times of 20 h (Ru/TiO_2_-1) reported earlier^6^. The conversion of the solid residue follows the same trend. The liquid product’s weight-average molecular weight (M_w_) (Fig. 4c, Supplementary Tables 9, 10) is lower over Ru/TiO_2_-8 and Ru/TiO_2_-12 than Ru/TiO_2_-1.

PP conversion follows^6^ (i) an initial polymer consumption forming a “heavy” liquid; (ii) a decrease of the liquid M_w_; and (iii) consumption of the liquid to light gases (~85% methane). When the M_w_ reaches a critical value, cascade hydrogenolysis to gas starts. Ru/TiO_2_-8 and Ru/TiO_2_-12 substantially increase the solid consumption and the liquid C–C bond hydrogenolysis compared to Ru/TiO_2_-1, leading to lighter products (Fig. 4c, Supplementary Tables 9, 10).

All catalysts show similar methane formation up to ca. 10% at long reaction times due to excessive liquid hydrogenolysis (Supplementary Figs. 24, 25, Supplementary Table 11). To study liquid gasification in more detail, we performed experiments at a higher polymer to catalyst ratio of 20 (Fig. 4b). The liquid stability increases in the order: Ru/TiO_2_-12 < Ru/TiO_2_-8 < Ru/TiO_2_-1 in line with the liquid M_w_.

In the PE conversion over Ru/ZrO_2_ and Ru/WO_x_/ZrO_2_, the hydrogen availability on Ru was crucial in controlling the hydrogenolysis selectivity to liquids vs. light gases^15^. A high intrinsic H coverage favors liquid products. Conversely, a low H coverage promotes a sequential cascade of C–C rupture to methane.

We hypothesize that the performance differences among catalysts stem from the H coverage on Ru. We perform experiments of varying H_2_ pressure (Fig. 4f). An increased hydrogen pressure leads to higher reaction rates over Ru/TiO_2_-1; the liquid yield reaches ca. 60% in 6 h (40 bar H_2_) and 3 h (50 bar H_2_), much faster than the 30 bar experiment. Still, the liquid to gas decomposition is also accelerated (Supplementary Fig. 26, Supplementary Table 12). This competition leads to a maximum liquid yield. The liquid decomposition on the ammonia-treated Ru/TiO_2_ samples is less severe, and a maximum is absent at a polymer to catalyst ratio of 20 (Fig. 4a). This maximum is visible at higher catalyst loadings (Fig. 4b, Supplementary Fig. 25) and shifts to shorter reaction times. The increased H coverage at the same H_2_ pressure drives the improved catalyst performance, consistent with the ^2^H MAS NMR data of the higher content of both Ru-H species and spillover (Fig. 2). We propose that the different nature of chemisorbed hydrogen may be partially responsible for the variation in the liquid yields over different samples. Thus, a simple increase in surface coverage of hydrogen is insufficient to get a similarly high liquid yield over Ru/TiO_2_-1 and Ru/TiO_2_-8.

**Fig. 5:**
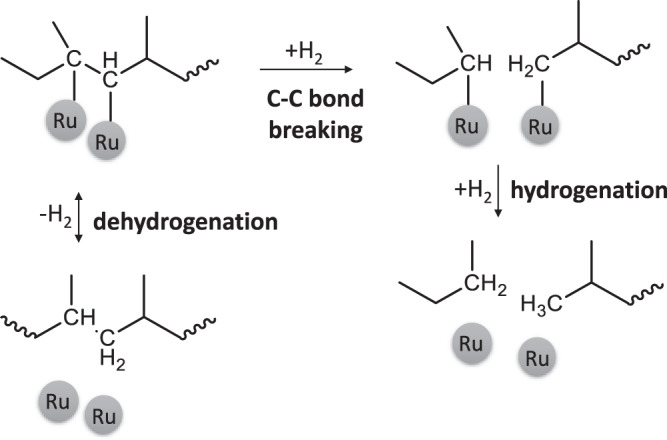
Major steps in C–C bond hydrogenolysis of polypropylene. Dehydrogenation happends in both directions, while C–C bond breaking is considered irreversible.

Alkane hydrogenolysis invokes (Fig. 5) adsorption to the metal, leading to dehydrogenated intermediates, C–C bond breaking of these intermediates, and final hydrogenation and product release. The first step is usually quasi-equilibrated^48^ due to the lower dehydrogenation barrier than the C–C bond breaking^49^. Experiments in D_2_ lead to slower dehydrogenation due to a kinetic isotope effect (KIE) in the dehydrogenation step, while the C–C bond breaking is not strongly affected by the H/D change^50^.

PP hydrogenolysis in D_2_ is slower^6^, and the M_w_ of the liquid is larger, consistent with our data on Ru/TiO_2_-1 and Ru/TiO_2_-8 (Fig. 4d, e). A prominent shift in M_w_ is evident over Ru/TiO_2_-1 (from 2.67 to 14.12 kDa). Quasi-stationary kinetic analysis with standard transition state theory calculations, based on Fig. 5 (see Supplementary discussion I, Supplementary Fig. 27), shows that increasing the H coverage increases the net hydrogenolysis rate, reduces the KIE, and makes the net reaction rate less sensitive to the H/D exchange. Since Ru/TiO_2_-8 has a higher H coverage, the reaction rate is less sensitive to deuteration and correlates with the hydrogenolysis data (Fig. 4a). Over the Ru/TiO_2_-8, a high H coverage pushes further C–C bond breaking, leading to consumption of the initial polymer with no solid residue. On Ru/TiO_2_-1, the lower H coverage makes dehydrogenation more kinetically relevant and the initial polymer deconstruction slower.

In PE conversion on Ru/ZrO_2_, higher H coverages accelerate the desorption of reaction intermediates, preventing them from over-cracking, and giving higher liquid selectivity over methane at high H_2_ pressures. In PP, an increased H coverage boosts all three reaction stages because the PP initial polymer deconstruction is slower than the product desorption. Thus, altering the H coverage primarily alters this reaction step.

## Discussion

Ru-based catalysts have demonstrated markedly different performances on various supports, but the reasons have remained elusive. For PE hydrogenolysis over Ru-WO_x_/ZrO_2_ catalysts^15^, a higher H surface coverage shifted the selectivity from terminal to internal C–C bond breaking, reducing methane formation at high hydrogen pressures. Partial reduction of the surface WO_x_ domains provided extra hydrogen storage, increasing H coverage. Hydrogen availability was hypothesized as important, but the generality of this concept and mechanistic insights leading to catalyst improvement have been lacking.

Here, we tuned the catalyst’s electronic properties while holding the Ru particle size and physical characteristics constant by modifying the synthesis using ammonia. Ammonia dehydroxylates the TiO_2_ surface reducing the density of Ti-OH groups. This, in turn, increases the Lewis acid strength of Ti^4+^ sites, evidenced by FTIR of CO, creating a more intimate contact of Ru and TiO_2_ with possible formation of a TiO_x_ peripheral layer, observed in previous reports^24^. Upon exposure to H_2_, the untreated (Ru/TiO_2_-1) sample shows moderate changes. H_2_ primarily dissociates and binds to Ru in the atop configuration, confirmed by ^2^H MAS NMR and DFT. Hydrogen spillover from Ru particles to the support leads to T^4+^ + $\bar{e}$→ Ti^3+^ reduction with subsequent electron trapping in the bandgap states. New Ti-OH groups form simultaneously, consistent with the classical spillover scheme (Fig. 3a).

For the NH_3_ treated samples (Ru/TiO_2_-8 and Ru/TiO_2_-12), spillover reduces TiO_2_ more extensively and forms delocalized $\bar{e}$ in the CB in addition to the shallow traps. Ru particles bind more hydrogen, not only covalently (Ru-H_atop_) but also through weak interactions involving Ru conduction electrons, revealed by ^2^H MAS NMR. This electron transfer from Ru to H leads to Ru^δ+^-H^**−**^ ion pairs as a new H binding mode on Ru. We speculate that TiO_2_ CB electrons created by spillover are responsible for activating this new pathway of hydrogen fixation on the Ru/TiO_2_ periphery.

Due to the higher H coverage on Ru, these catalysts show higher activity in PP hydrogenolysis leading to higher liquid yields and doing so in a shorter time (6 vs. 16 h). Comparison with previously reported PP hydrogenolysis results (Supplementary Table 13) shows that Ru/TiO_2_-8 provides higher liquid yields at shorter reaction times than Ru/TiO_2_-1 and Ru/C. Ru/CeO_2_ shows higher liquid yields (83 vs. 74.1%) at higher catalyst loading and longer reaction times, which make a more detailed comparison hard. A similar acceleration is achieved by increasing the hydrogen pressure. High surface H coverage is manifested in a reduced KIE upon H_2_/D_2_ substitution.

Finally, we proposed a simple way to tune metal-support interactions in Ru/TiO_2_ by adding NH_3_ as a pH modulator in the Ru deposition step. We demonstrated a boost in hydrogen storage capacity stemming from a pronounced H spillover from Ru to TiO_2_. ^2^H MAS NMR proved to be a sensitive, semi-quantitative tool to study hydrogen chemisorption. Coupled with in situ pretreatment and heating, it can provide direct quantitative monitoring of hydrogen species on metal-metal oxide interfaces. Further improvements to the methodology could expand its scope. Raman, FTIR, and NAP-XPS highlight the enhanced spillover is caused by electrons directly filling the conduction bund of TiO_2_ compared to the standard localized Ti^3+^ trap sites. This enhances the hydrogen binding capacity for Ru particles due to the relatively weak charge transfer. Technologically, a higher hydrogen coverage increases the liquid yield to ~74% just in 6 h vs. 63% in ~20 h over the conventional catalyst possessing less pronounced MSI. The hydrogen availability drives catalyst improvement and emerges as a general strategy for designing catalysts beyond Ru/TiO_2_ and PP hydrogenolysis.

## Methods

### Catalyst preparation

The Ru/TiO_2_ catalysts were prepared by wetness impregnation using a commercially available anatase TiO_2_ support (US Research Nanomaterials). Before impregnation, the TiO_2_ powder was calcined at 450 °C for 6 h in static air. Ru/TiO_2_-1 was prepared by mixing 2.77 g of Ru precursor solution (ruthenium(III) nitrosyl nitrate solution in dilute nitric acid, Sigma–Aldrich) with 1 g of deionized water and then adding it to TiO_2_ powder under manual stirring with a glass rod at 70 °C. For Ru/TiO_2_-8 and Ru/TiO_2_-12, the pH was adjusted accordingly by adding several drops of aqueous NH_3_ (25%, Supelco). After impregnation, the catalyst was dried at 100 °C overnight and reduced in H_2_ (50% in He) flow in a tubular furnace at 300 °C for 2 h (ramp rate 10 °C/min).

### Catalyst characterization

The Ru loading was estimated using X-ray fluorescence (XRF) analysis on a Rigaku Supermini 200 WDXRF in a He atmosphere. XRD (X-ray powder diffraction) patterns were obtained on a Bruker D8 diffractometer with 0.05° 2θ step size using Cu Kα radiation (λ 1.54 Å). Weight vs. temperature curves was recorded on a Discovery TGA instrument in a flow of 5%H_2_/He with 10 °C/min ramp from 30 to 600 °C. N_2_ sorption isotherms at −196 °C were recorded on a Micromeritics ASAP 2020 instrument. Before measurements, the samples were degassed at 300 °C for 3 h. UV-vis spectra were collected in diffuse-reflectance mode on a spectrometer (JASCO, V-550) with a diffuse-reflectance attachment using an ambient conditions cell with a quartz window and BaSO_4_ as a standard. The optical bandgap was estimated using the Tauc plot of (*ahv*)^2^ vs. *hv*, where *hv* is the photon energy in eV, and *α* is the reflectance. In-house X-ray photoelectron spectra (XPS) were recorded on a Thermo Fisher K-Alpha+ machine with an Al Kα monochromatic source. Before measurements, the samples were reduced in 50% H_2_/He flow at 300 °C and then deposited on a Cu foil. For binding energy reference, the C 1 s line at 284.6 eV was used. Scanning transmission electron microscopy (STEM) images were acquired on an Aberration Corrected Scanning/Transmission Electron Microscope, JEOL NEOARM TEM/STEM.

Temperature-programmed desorption (TPD) of H_2_ was recorded on a Micromeritics Autochem II instrument. Approximately 0.2 g of samples were packed in a U-shaped quartz reactor, heated in 10%H_2_/Ar flow (50 ml/min) to 300 °C with 2 h dwell time. Then samples were cooled to 35 °C in the same 10%H_2_/Ar flow, then purged isothermally for 30 min with pure Ar. Afterward, samples were heated with 10 °C/min ramping rate to 700 °C in Ar flow with an online thermal conductivity detector (TCD) recording of hydrogen desorption. Temperature-programmed reduction (TPR) with H_2_ was recorded on the same Autochem II instrument. Samples were pre-reduced in 10%H_2_/Ar (50 ml/min) flow to 300 °C with 2 h dwell time. Then samples were cooled to 35 °C in the same 10%H_2_/Ar flow and then heated with 10 °C/min ramping rate to 700 °C with an online TCD recording of hydrogen consumption. Hydrogen pulse chemisorption at 35 °C was measured on the same instrument. Samples were pretreated in 10%H_2_/Ar flow (50 ml/min) to 300 °C with 2 h dwell time and then for 1 h in pure Ar at the same temperature. Then, the reactor was cooled to 35 °C, and hydrogen was pulsed using 6-port valve with 45 μmol H_2_ per pulse, calibrated with the empty reactor.

Fourier transform infrared spectroscopy (FTIR) spectra of adsorbed CO at −196 °C were measured on a Nicolet 8700 spectrometer equipped with liquid nitrogen cooling and an MCT detector. The sample was pressed into a self-supported wafer (64 bar/in^2^) and loaded into a homemade quartz cell connected to the vacuum line. The sample was pretreated at 300 °C for 1 h in a vacuum (<10^−4^ torr). Then the sample was exposed to 1 torr of H_2_ gas for 30 min and evacuated for 10 min to reduce Ru. The reduction was repeated four times, followed by a final evacuation for 30 min. Afterward, the sample was cooled to −196 °С and exposed to 1 torr CO to saturate the surface. Then the CO was evacuated till 10^−3^ torr with spectra measured in ~0.1 torr increments.

Diffuse-reflectance infrared Fourier transform spectroscopy (DRIFT) spectra, which are more sensitive than FTIR to surface OH groups^51^, were recorded on the same spectrometer with a Praying Mantis attachment and in situ Harrick cell. The samples were reduced in 50%H_2_/He flow for 2 h at 300 °C followed by flushing with pure He at 300 °C for 30 min. Then the samples were cooled to 35 °C, and DRIFTS spectra were recorded.

### FTIR and Raman spectroscopy of hydrogen spillover

Samples were pressed in self-supporting wafers (64 bar/in^2^) and placed in a homemade transmission IR cell connected to an atmospheric pressure gas flow manifold. Samples were pre-reduced at 300 °C for 2 h in 100 ml flow of 20%H_2_/He mixture (ramp rate 10 °C/min). Then, the samples were flushed with pure 100 ml/min He flows for 1 h and cooled to treatment temperature (200 or 250 °C). A baseline spectrum was recorded in pure He, and then the gas flow was switched from He to x%H_2_/He with a hydrogen partial pressure (p(H_2_)) in the 0.06–0.14 bar range. Spectra were recorded in 10 s increments until complete saturation at a given p(H_2_) and temperature (~15 mins). Then the baseline spectrum was subtracted to highlight the spillover effect.

Raman spectra were measured on a Horiba LabRAM HR evolution spectrometer using 532 nm green laser (5 mW power), ×50 objective, and 1800 g/mm grating. The detector resolution under these conditions is equal to 0.48 cm^−1^/pixel. In situ measurements were done using a Harrick Raman cell equipped with a quartz window. Samples were pre-reduced at 300 °C for 2 h in 50 ml flow of 20% H_2_/He mixture (ramp rate 10°/min). Then, samples were flushed in pure 50 ml/min He flows for 1 h and cooled to treatment temperature (200 or 250 °C). The baseline spectrum was recorded in pure He, and then the gas flow was switched from He to 15%H_2_/He. Spectra were recorded after 10 min stabilization to ensure completion of spillover. Spectra were baseline corrected and fitted using the Lorentzian function in the Omnic software.

### ^2^H MAS NMR spectroscopy of chemisorbed deuterium

The sample was packed in ZrO_2_ 4 mm standard MAS NMR rotor, loaded in a pyrex tube with a valve, and connected to a vacuum line. Then it was heated to 120 °C for 1 h to remove moisture and then to 300 °C for 3 h under 10^−4^ torr. Then, 1 torr of D_2_ gas (99.8%, Cambridge Isotope Laboratories, Inc.) was administered through the vacuum line. After reduction for 30 min, the sample was evacuated for 30 min to remove possible traces of water. The treatment with D_2_ at 300 °C was repeated four times. Then the sample was cooled to room temperature under 1 torr D_2_ and transferred to a glove box without exposure to air. In nitrogen, the glove box rotor was removed from the tube and sealed. To test the stability of chemisorbed deuterium, in an experiment, the sample was degassed at 100 °C for 30 min at 10^−4^ torr and only then sealed.

^2^H MAS NMR spectra were acquired on an 11.7 T Bruker Avance III NMR spectrometer with a 4 mm HX probe at a ^2^H frequency of 76.77 MHz at magic angle spinning speeds of 4 and 10 kHz. The samples were maintained at room temperature with sample heating due to magic angle spinning taken into account. Data were acquired using a 180°-τ−90° pulse sequence with an interpulse delay of 10 ms to reduce baseline distortions, with a 90° pulse length of 4.25 μs. 16,384–20,480 scans were collected per sample with a pulse delay of 4 s. Spectra were referenced externally to D_2_O at 4.7 ppm.

Spectra were modeled and fitted using the ssNake software^52^, and three component refinement of the experimental spectra was performed to estimate the key EFG parameters.

### Near ambient pressure X-ray photoelectron spectroscopy (NAP-XPS)

The NAP-XPS spectra were collected under UHV (base pressure of 10^−9^ mbar) using a SPECS electron spectrometer equipped with PHOIBOS 100 hemispherical energy analyzer and a monochromatic Al Kα X-ray source (1486.7 eV). Spectra were referenced to C*1s* line at 284.6 eV and fitted using Thermo Avantage software. The samples were pressed on a Cu piece and mounted on a stainless-steel flag-type holder with a K-type thermocouple allowing for in situ temperature readings. After loading the sample in XPS analysis chamber, it was heated to 300 °C and kept at that temperature for 30 min (ramp rate 10°/min). Then 1 mbar hydrogen was dosed for in situ reductions at 300 °C for 40 min. Then hydrogen was evacuated, and the sample was annealed at 300 °C in UHV for 10 min to remove chemisorbed hydrogen. Afterward, the sample was cooled to 200 °C, and initial spectra in UHV were recorded. Then the sample was exposed to 1 mbar hydrogen for 15 min at the same temperature and new spectra were measured. Additional reference spectra were recorded also at 35 °C in UHV.

### Reaction tests

Isotactic polypropylene (PP, M_w_ ~250,000, M_n_ ~67,000) was purchased from Sigma–Aldrich. An amount of freshly reduced catalyst (0.1 or 0.05 g) was mechanically mixed with 2.0 g of PP using a vortex mixer. The mixture was then transferred into a borosilicate liner of a 50 mL stainless-steel Parr reactor with a 0.7 mL stir bar. The mass ratio of polymer to catalyst was 20 or 40, corresponding to the catalyst loading of 0.1 or 0.05 g. The Parr reactor was sealed and purged six times with pure H_2_ at 50 bar, charged to 30 bar, and then heated to 250 °C (ramping rate 10 °C/min) using a band heater (Omega Eng.). Stirring was initiated after the temperature reached 160 ± 5 °C to first melt the polymer. The stirring speed was set at 300 rpm; additional experiments showed similar product yields and molecular weight distributions at 400 and 500 rpm but nearly no reactivity without stirring. Reactions were maintained for specified time intervals and then quickly quenched in an ice bath. For H_2_ pressure variation experiments, the hydrogen pressure was 20, 40, and 50 bar.

### Product analysis

After the temperature dropped below 10 °C, the gas from the reactor’s headspace was transferred to a 1 L Tedlar gas sampling bag for analysis. Then the reactor was opened, and the liquid and solid residue were mixed with 20 mL of CH_2_Cl_2_, used as a solvent. This slurry was filtered (Whatman, 100 μm), and the solid residue was dried at room temperature overnight with complete evaporation of all CH_2_Cl_2_. The solvent was removed from the liquid fraction using a rotary evaporator. The solid and liquid fraction yields were quantified gravimetrically. A GC with an FID detector (Agilent 7890 Series, HP-volamine column) was used for gas analysis. The concentration of hydrocarbons in the gas sample was calculated using a standard C_1_–C_4_ calibration mixture. The absolute amount of hydrocarbons in the gas was calculated using the ideal gas law.

An overall balance was calculated according to Eq. (1):

1 $${{{{{{\mathrm{Material}}}}}}\; {{{{{\mathrm{balance}}}}}}}=\frac{{m}_{L}+{m}_{s}+{m}_{g}}{{m}_{{{{{{{\mathrm{initial}}}}}}}}}\cdot 100\%$$

where ${m}_{L},{m}_{s},{m}_{g},{m}_{{{{{{{\mathrm{initial}}}}}}}}$ are the mass of liquid, gas, solid, and initial polymer, respectively. The yield of the i-th group of products was calculated according to Eq. (2):

2 $${Y}_{i}=\frac{{m}_{i}}{{m}_{{{{{{{\mathrm{initial}}}}}}}}}\cdot 100\%$$

where *m*_*i*_ is the mass of the ith group of products (liquid, gas, or solid).

Liquid products were analyzed using gel permeation chromatography (GPC) using Styragel HR 4, HR 3, and HR 0.5 columns (dimensions 4.6 × 300 mm) connected in tandem using THF as solvent (0.3 ml/min flow rate) and a Waters 2414 refractive index detector (RID). The retention time was calibrated using Polystyrene Standards Kit (Waters, WAT058931).

### Density functional theory (DFT) calculations

The TiO_2_ support was modeled as an anatase structure with the (101) facet exposed. The supported Ru was modeled as a Ru-12 cluster on the TiO_2_ surface. DFT calculations were performed using the Vienna Ab initio Simulation Package (VASP) package^53^. The RPBE potential was used in conjunction with the D3 correction^54^. The energy cutoff was set as 520 eV, and the electron smearing factor was set as 0.1 eV. Electronic structures were convergence until 10^−6^ eV, and ionic steps were iteratively taken until the force fell below 0.05 eV/A. The Electric Field Gradient (EFG) was calculated using the VASP internal functions. Nuclear quadrupole moments were adapted accordingly^55^.

## Supplementary information

Supplementary Information

## Data Availability

All data generated or analyszed during this study are included in this published article (and its supplementary information file).
