# Supplementary material for: Electronic modulation of metal-support interactions improves polypropylene hydrogenolysis over ruthenium catalysts
Source: Nat Commun. 2022 Sep 3;13:5186. doi: 10.1038/s41467-022-32934-5 (PMC9440920; doi:10.1038/s41467-022-32934-5)
Supplement: Supplementary file 1 — Supplementary Information [file 41467_2022_32934_MOESM1_ESM.pdf]

Supporting information for:

Electronic modulation of metal-support interactions improves polypropylene hydrogenolysis over ruthenium catalysts

Pavel A. Kots[1], Tianjun Xie[1], Brandon C. Vance[1,2], Caitlin M. Quinn[3], Matheus Dorneles de Mello[4], J. Anibal Boscoboinik[4], Cong Wang[1], Pawan Kumar[5], Eric A. Stach[5], Nebojsa S. Marinkovic[6], Lu Ma[7], Steven N. Ehrlich[7], Dionisios G. Vlachos[1,2*]

[1]Center for Plastics Innovation, University of Delaware, 221 Academy St., Newark, DE 19716, USA

[2]Department of Chemical and Biomolecular Engineering, University of Delaware, 150 Academy St., Newark, DE 19716, USA

[3]Department of Chemistry and Biochemistry, University of Delaware, Newark, DE 19716, USA

[4]Center for Functional Nanomaterials, Brookhaven National Laboratory, 735 Brookhaven Ave, Upton, NY 11973, USA

[5]Department of Materials Science and Engineering, University of Pennsylvania, Philadelphia, PA 19104, USA

[6]Department of Chemical Engineering, Columbia University, 500 W 120th St., New York, NY 10027, USA

[7]National Synchrotron Light Source II, Brookhaven National Laboratory, Upton, NY 11973, USA

*Corresponding author. Email: vlachos@udel.edu.

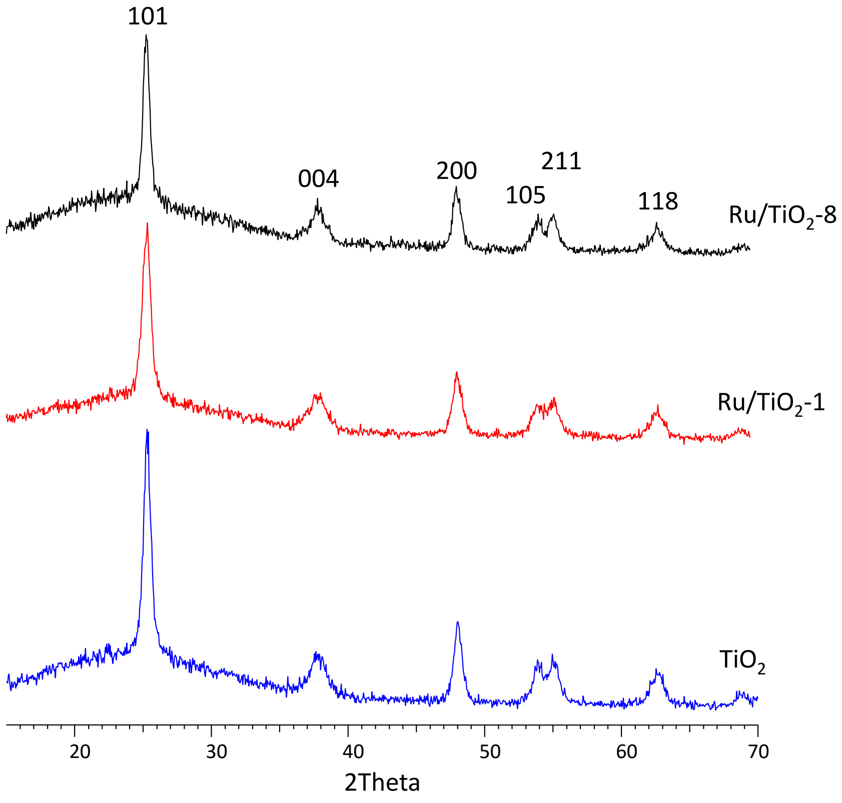

Supplementary Figure 1. **XRD patterns of Ru/TiO_2 samples.**

XRD patterns show that samples contain pure TiO_2 anatase with similar crystal sizes. There is no sign of Ru crystals due to the small Ru particle size.

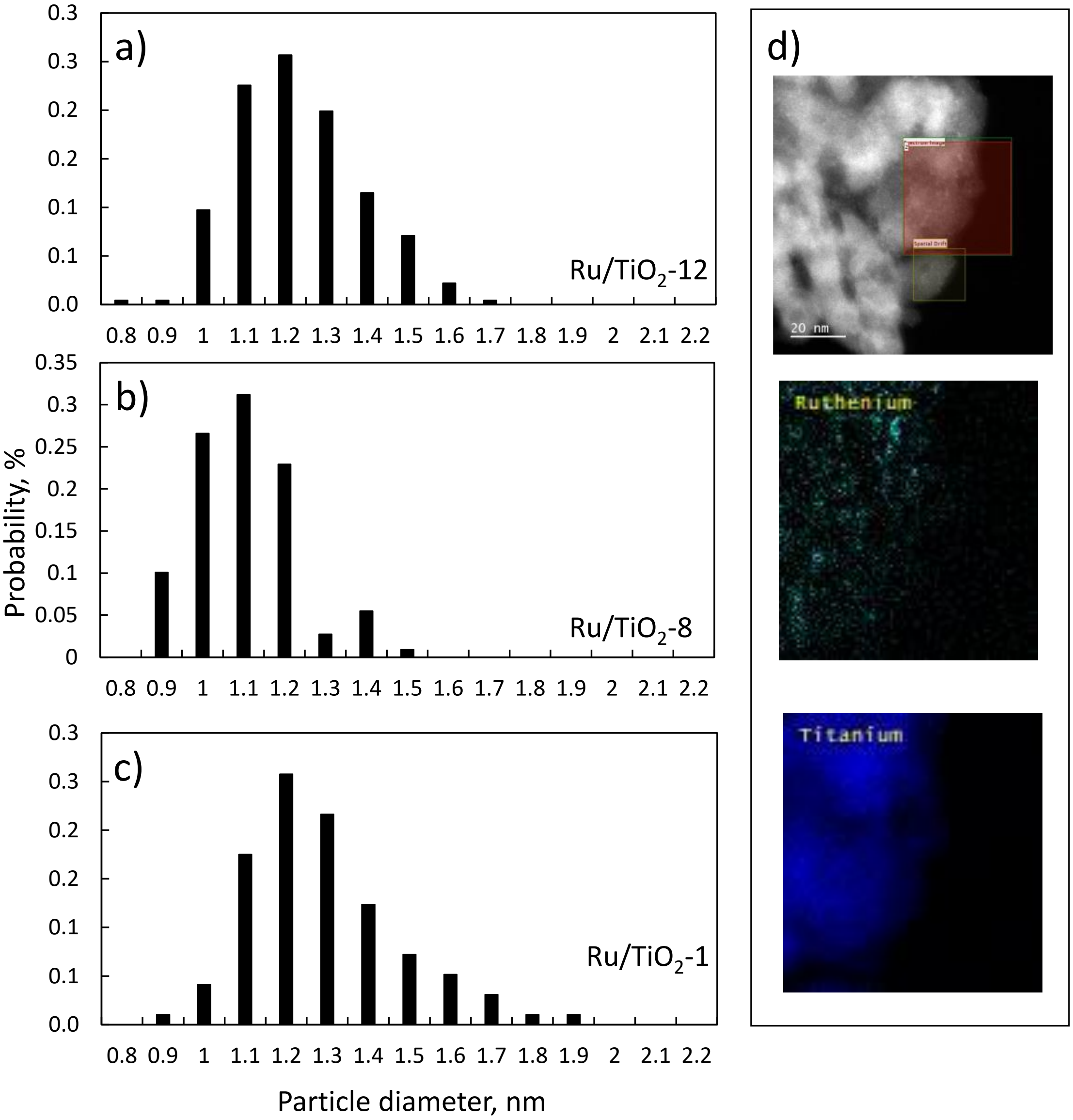

Supplementary Figure 2. **Particle size distribution derived from STEM images. a,** Ru/TiO_2-1. **b,** Ru/TiO_2-8. **c,** Ru/TiO_2-12 samples. **d,** EDX map of a selected area of Ru/TiO_2-12 sample.

All samples show very similar particle size distribution, with Ru/TiO2-8 having slightly smaller particles (1.1 vs 1.2-1.3 nm). EDX maps confirm the STEM data.

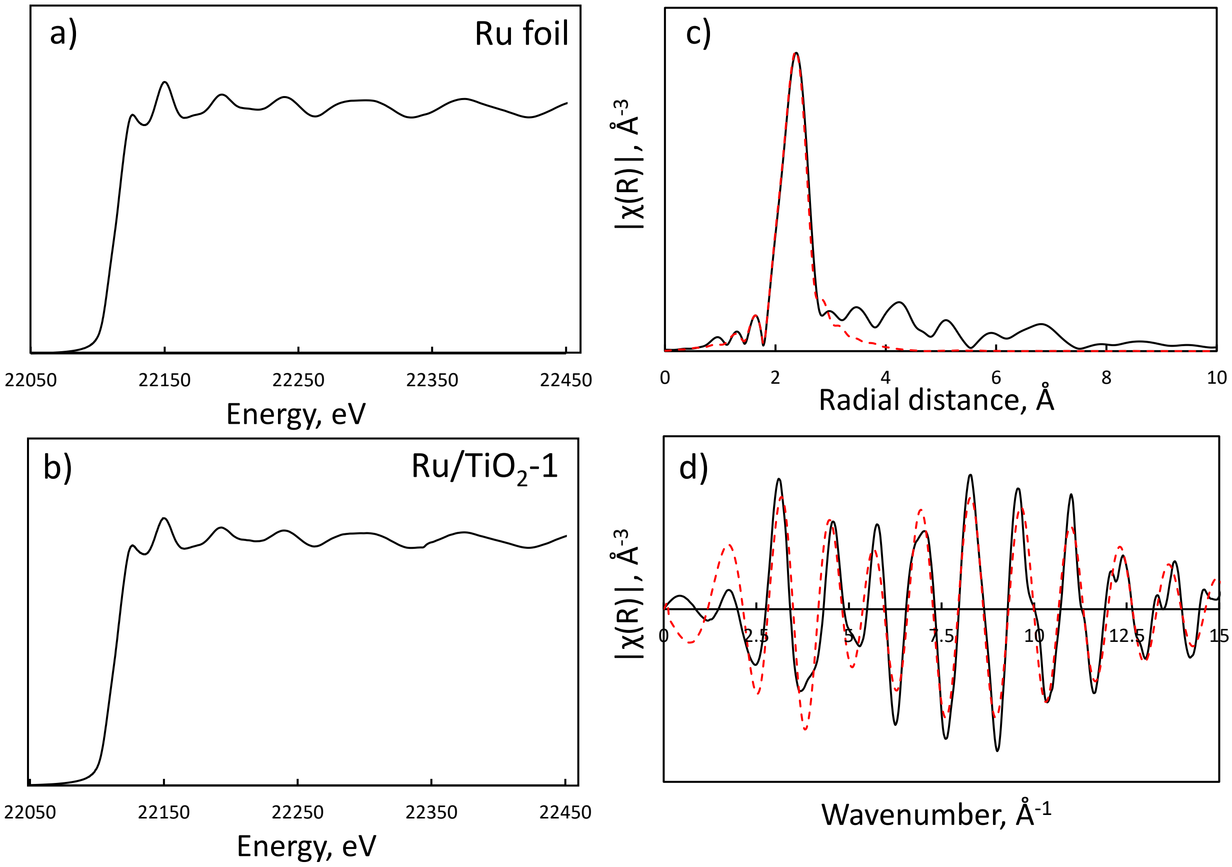

Supplementary Figure 3. **XANES spectra**. **a,** Standard Ru foil; **b,** Ru/TiO_2-1 sample. **c,d,** Quality of EXAFS data fitting using parameters from Table S1 in R-space (**c**) and in k-space (**d**), respectively.

Supplementary Table 1. Ru's structural parameters and corresponding Ru particle size.

Sample	CN[a]	DWF, 10-3 $Å^2$ [b]	R(Ru-Ru), Å[c]	E_0, eV[d]	d(Ru), nm[e]
Ru foil standard	12	3.3	2.67	0.06	
Ru/TiO_2-1	8.5	1.3	2.67	-1.68	1.4
Ru/TiO_2-8	8.6	1.8	2.66	-1.62	1.4

[a]Ru-Ru first shell coordination number; [b]Debye-Waller factor; [c]Ru-Ru first shell bond distance; [d]Energy reference; [e]Ru particle diameter.

Using Eq. (7) of Ref.[1], we calculated particle size using CN extracted from EXAFS data. This value is an estimate since Ru particles can have a 2D nanoslab shape rather than a spherical one.[2]

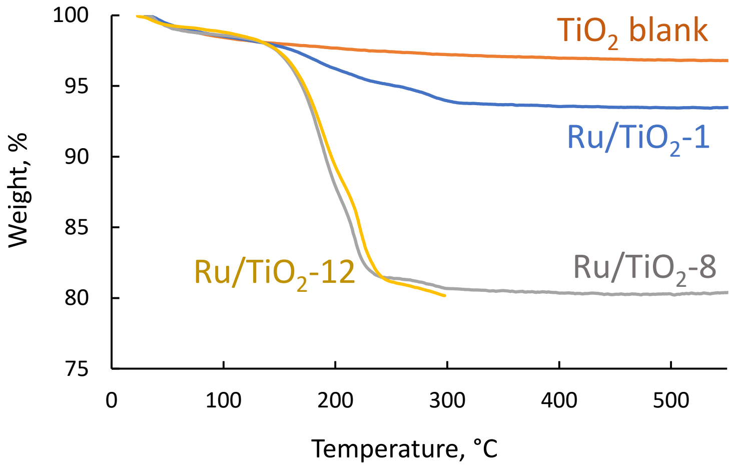

Supplementary Figure 4. **TGA curves**. Measured in 10% H_2/He flow during the initial reduction of Ru/TiO_2 samples after impregnation with the Ru precursor and drying.

TGA curves show that NH_3 is retained after the impregnation in large quantities and is removed at high temperatures. Ammonia treatment is known to induce changes in the electronic structure of TiO_2 since it is a reductant increasing the localized Ti^{3+} sites.[3]

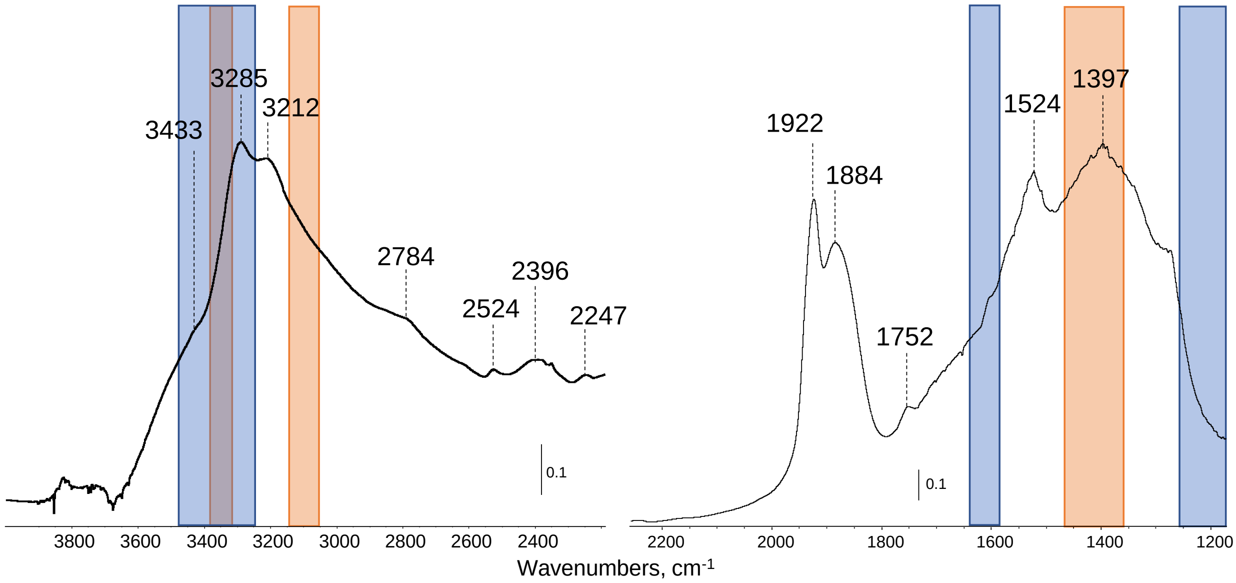

Supplementary Figure 5. **Difference DRIFT spectra of Ru/TiO_2-8 at 100 °C.** Blue bars show bands tentatively assigned to NH_3, red – to NH_4^+. Spectrum of NH_3-free Ru/TiO_2-1 at 100 °C was used for subtraction. Gas flow: 50%H_2/He, 20 ml/min.

The DRIFT spectra of Ru/TiO_2-8 sample before steep ammonia desorption show a very broad set of peaks in the 3520-2650 cm^{-1} range due to various NH stretching vibrations. Clearly, adsorption of ammonia produces several types of surface species. The presence of coordinatively bond NH_3 is evident by the peaks at ca. 3445 cm^{-1} (asymmetric NH stretching ν_3 mode) and 3340 cm^{-1} (symmetric NH stretching ν_1 mode)[4]. Ammonium cation should give rise to peaks at 3300 cm^{-1} due to the asymmetric NH stretching.[5] The spectrum in the low-frequency region provides further evidence of the co-existence

of coordinated ammonia and ammonium cations. Peaks at ~1600 and 1225 cm^{-1} correspond to NH_3,[5] while the broad signal at 1397 cm^{-1} arises from the bending mode of NH_4^+ with overtone at 2784 cm^{-1}. The signal at 1524 cm^{-1} is assigned to NH_3 in the Ru^{3+} coordination sphere.[6] The peaks at 1922, 1884, and 1752 cm^{-1} are due to NO ligands.

The overall shape of the spectrum in Supplementary Figure X is not typical for pure NH_3 adsorbed on anatase.[7] The dissociation of NH_3 on TiO_2 surface produces NH_2 species with typical bands at 3350, 3190, and 1220 cm^{-1}.[8] Also the bands at 1610-1560 cm^{-1} (NH_2 scissoring), 1350-1300 cm^{-1} (NH_2 wagging) should be present, according to other reports.[7] The formation of NH_x groups is known on bare anatase surface and is facilitated by the presence of Ru cations or nanoparticles. Due to the broadening of the peaks, it is impossible to differentiate NH_2 groups from coordinated NH_3 or NH_4^+.

Dehydroxylation facilitated by ammonia can occur through a proton transfer mechanism depicted in Supplementary Figure 6.

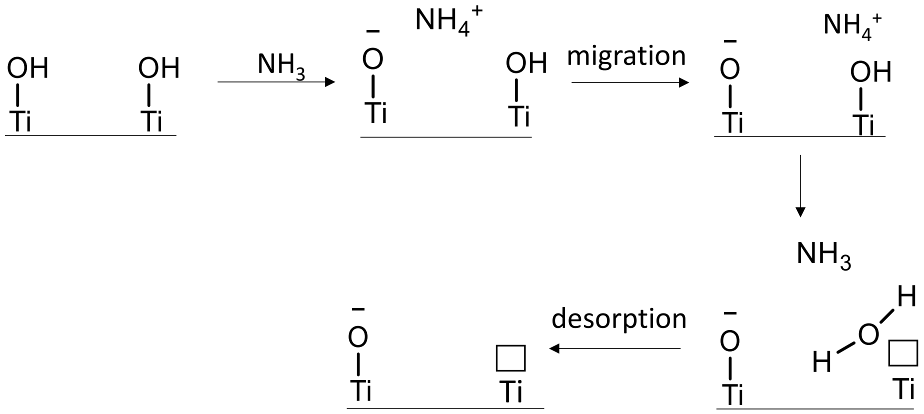

Supplementary Figure 6. **Proton transfer mechanism of ammonia-facilitated dehydroxylation of TiO_2**.

Two distant OH groups on the TiO_2 surface can be dehydroxylated with NH_3 serving as a proton transfer catalyst. After the initial protonation, NH_4^+ forms on one OH group, and then NH_4^+ diffuses to the second OH group. After proton transfer, water forms and then both NH_3 and H_2O desorb, leaving reduced Ti-O^- and an anion vacancy. Thus, the presence of NH_3 facilitates dehydroxylation of two OH groups, which would not have happened thermally due to the distance between them.

Another pathway for dehydroxylation involves partial dehydrogenation of NH_3 over TiO_2 with the formation of NH_x species.

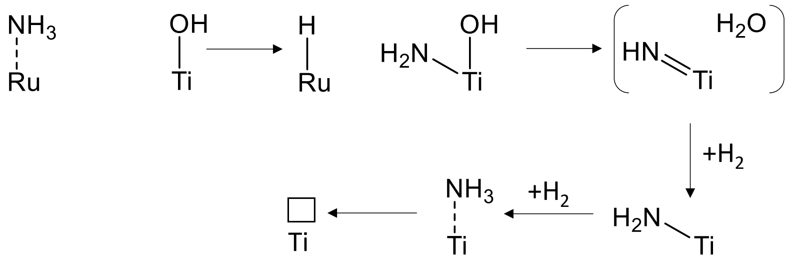

Supplementary Figure 7. **NH_x-mediated mechanism of TiO_2 dehydroxylation.**

Ammonia can be dehydrogenated on bare TiO_2 or over Ru^{3+}/Ru^0 sites leading to NH_2 groups. These can dimerize to N_2H_4 hydrazine, known as strong reductant or act directly as NH_2 monomers.[5] These groups interact with neighboring OH groups where they can reduce Ti^{4+} cation (depicted on Supplementary Figure 7, as formation of Ti=N bond) and liberate water. Later, hydrogen supplied from the Ru nanoparticles will reduce Ti=NH imides to amines and ultimately to NH_3, leaving a reduced Ti center on the surface. This mechanism was proposed previously to explain the reduction of metal cations on TiO_2 by NH_2 groups.[7] It shows that the formation of NH_x species on the surface could lead to both dehydroxylation and partial reduction of TiO_2.

In order to check whether TiO_2 is partially reduced by NH_3 during catalyst preparation, we performed reduction of impregnated samples in H_2/He flow mimicking catalyst preparation followed by *in situ* Raman spectroscopy (Supplementary Figure 6).

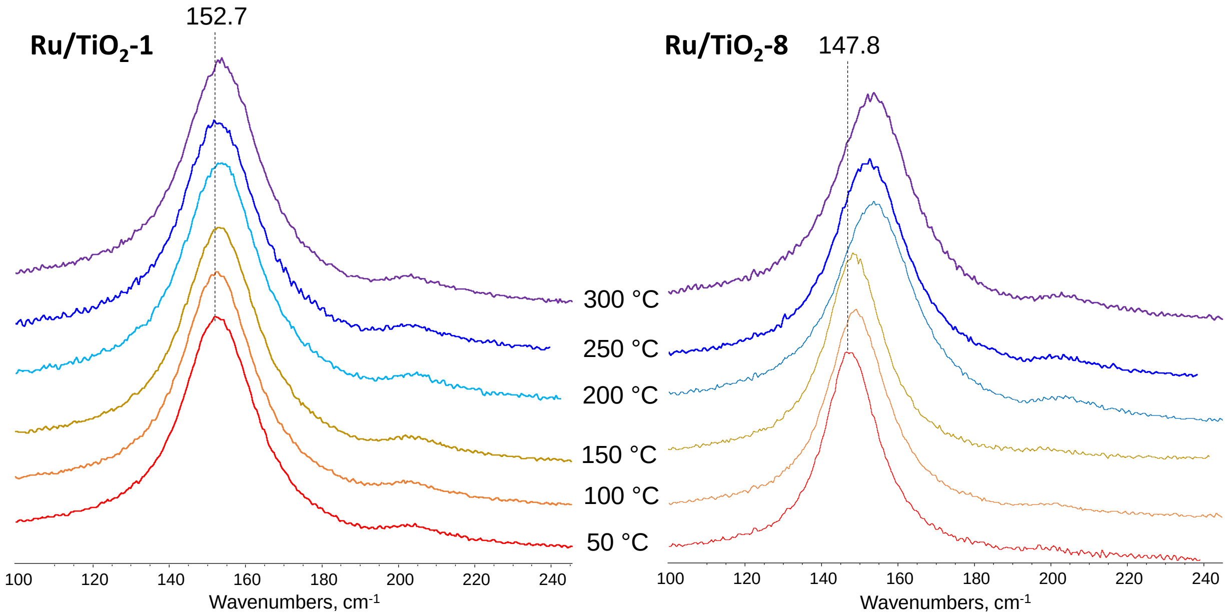

Supplementary Figure 8. **Raman spectra of impregnated Ru/TiO_2-1 and Ru/TiO_2-8 samples heated in 50% H_2/He flow.**

The $E_g(1)$ vibrational mode of TiO_2 anatase (~144 cm^{-1}) is sensitive to the concentration of charge carriers in the framework.[9] The peak position is used to indirectly monitor the progress of TiO_2 reduction. For Ru/TiO_2-1, the peak shifts slightly from 152.7 to 153.4 cm^{-1} when the reduction temperature increases from 50 °C (initial sample) to 300 °C (full reduction). For the Ru/TiO_2-8 sample, the peak shifts slightly

from 147.8 to 148.7 cm^{-1} in 50-150 °C range. Then $E_g(1)$ mode reaches 153.4 cm^{-1} at a higher reduction temperature. The jump in $E_g(1)$ peak position coincides with the massive NH_3 desorption from the sample surface. It appears that partial reduction of TiO_2 (the high-frequency peak shift) is connected with the desorption of ammonia.

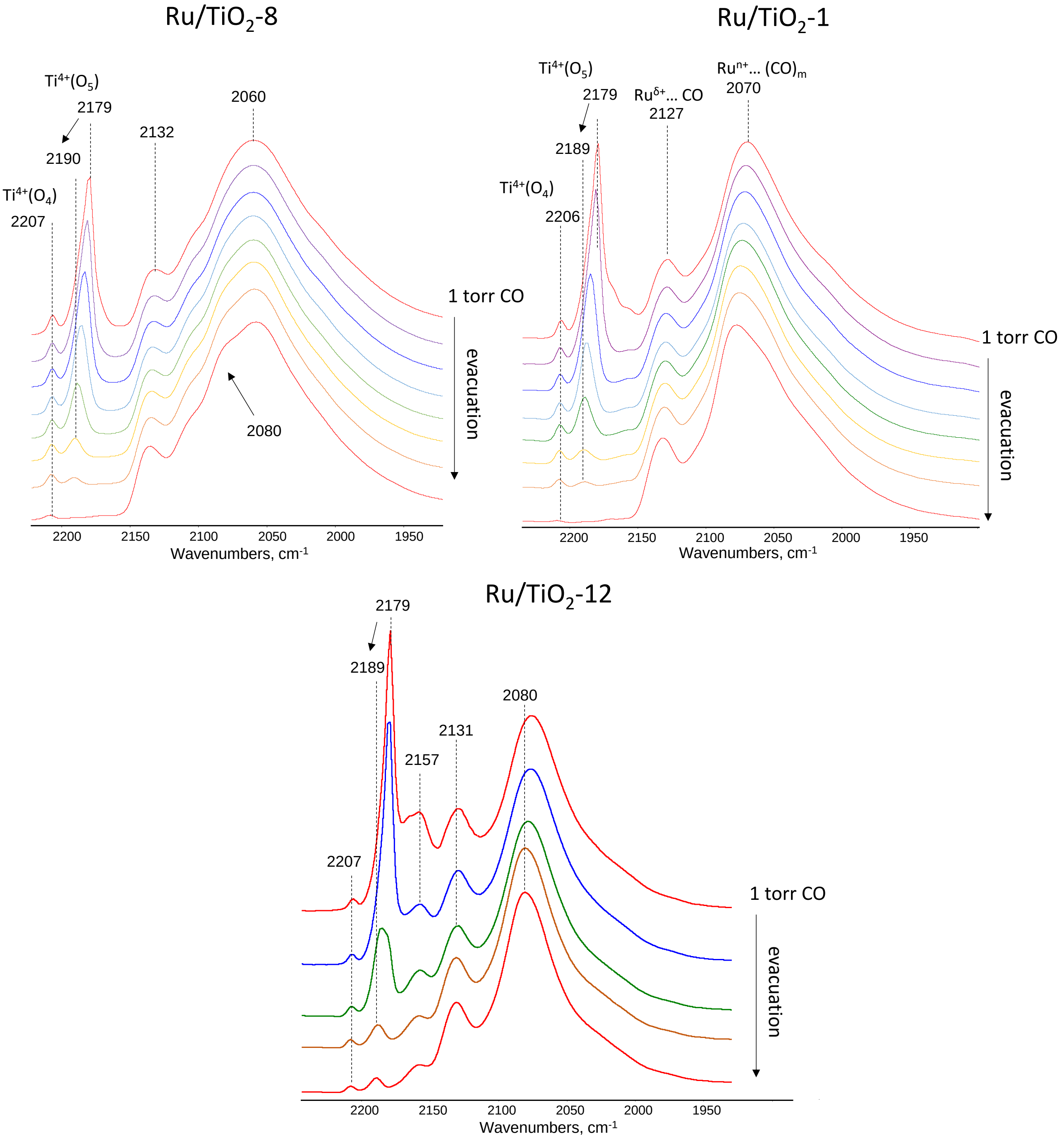

Supplementary Figure 9. **FTIR spectra of CO chemisorbed on Ru/TiO_2 samples at -196 °C**. Spectra from top to bottom show the progress of CO desorption. $Ti^{4+}(O_5)$ – pentacoordinated Ti^{4+} ion, $Ti^{4+}(O_4)$ – tetracoordinated Ti^{4+} ion.

Supplementary Figure 9 shows that each sample gives bands due to CO bonded to Ti^{4+} Lewis acid sites (2179 and 2207 cm^{-1}), and CO in ionic Ru-carbonyls (the broad band in the 2150-1900 cm^{-1} region). During CO evacuation at 77K, CO is removed completely from the Ru/TiO_2-1. For Ru/TiO_2-8 and Ru/TiO_2-12, both Ti^{4+}…CO bands at 2207 and 2190 cm^{-1} are still present after 10 min evacuation, indicating stronger Lewis acid sites than on Ru/TiO_2-1. The broad band of Ru-CO carbonyls is a superposition of several overlapping bands due to $Ru^{n+}(CO)_m$ multicarbonyl species originating from CO-induced oxidation of Ru nanoparticles.[10] Analysis using the second derivative (Supplementary Figure 11) shows distinctive bands at 2136-2131, 2107, and 2084 cm^{-1} for all samples. For Ru/TiO_2-1 and Ru/TiO_2-8, an extra band at 2055-2053 cm^{-1} is probably due to $Ru^{3+}(CO)_2$.[11] No other bands at ~2040 cm^{-1} corresponding to CO bonded to metallic Ru[12] were observed. Ru particles are oxidized by CO almost completely, forming various multicarbonyls. Interestingly, peak maxima and relative intensity differ among samples due to variation in metal-support interactions.

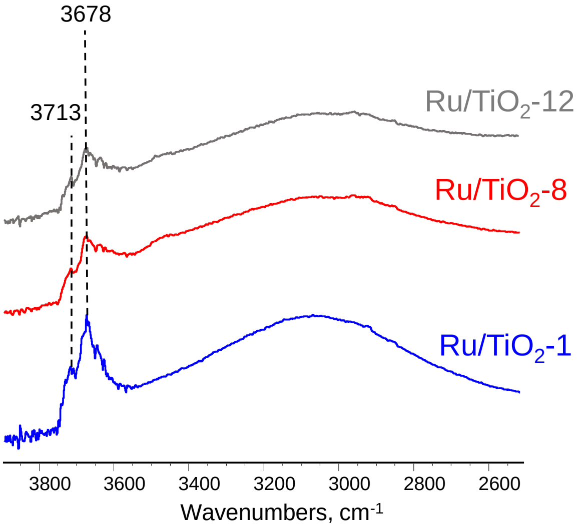

Supplementary Figure 10. **DRIFT spectra of Ru/TiO_2 samples at 35 °C after pretreatment at 300 °C in H_2.**

Both 3713 and 3678 cm^{-1} bands are typical ν(OH) vibrations of anatase Ti-OH groups.[13] Shoulder at ~3635 cm^{-1} is assigned to the bridging OH group on the anatase surface.[14]

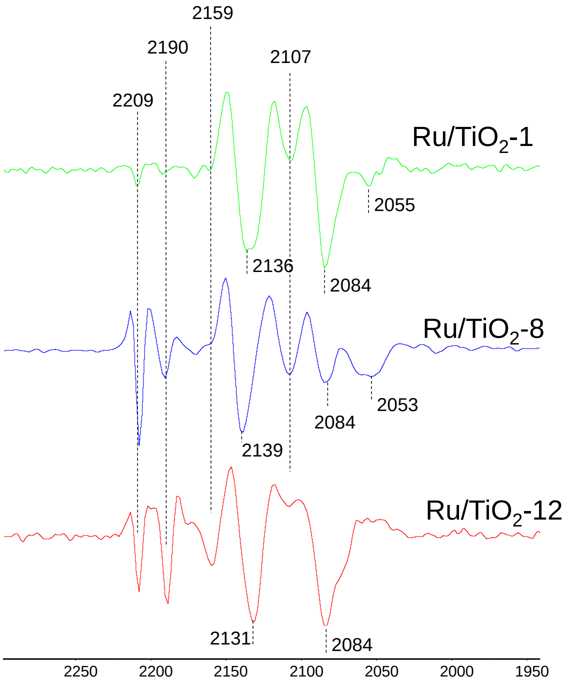

Supplementary Figure 11. **Second derivative of FTIR spectra of adsorbed CO at -196 °C over Ru/TiO_2 samples**. Measured after 10-minute evacuation (pressure <7·10^{-3} bar).

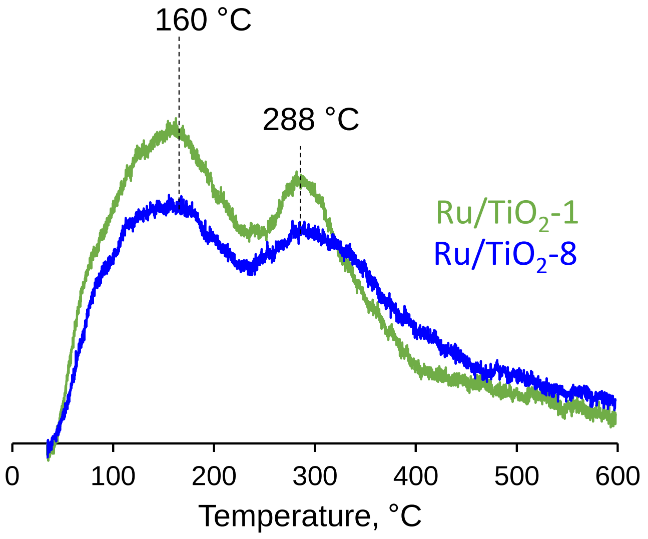

Supplementary Figure 12. **TPD curves of Ru/TiO_2 samples**.

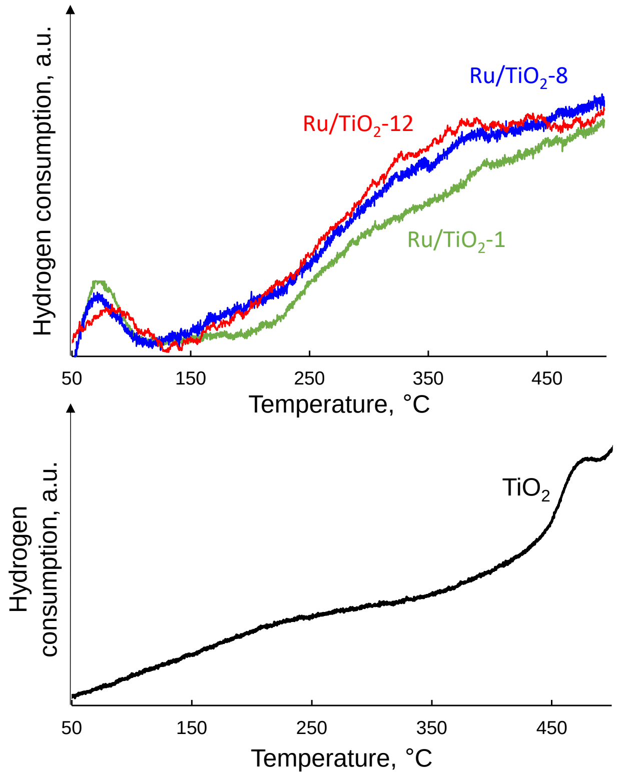

Supplementary Figure 13. **Hydrogen TPR curves of Ru/TiO_2 samples and pure support**.

^2H MAS NMR spectroscopy

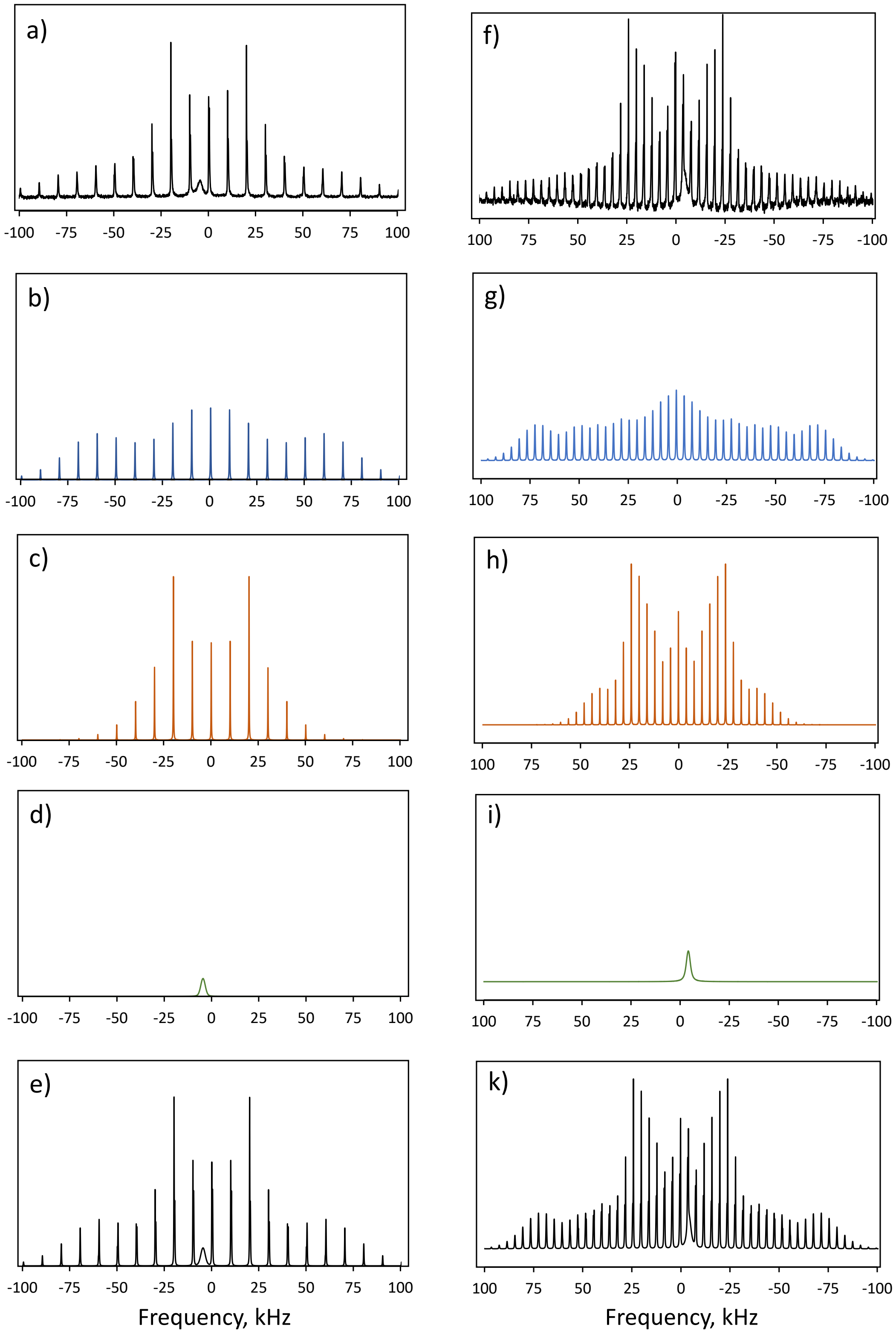

Supplementary Figure 14. **^2H MAS NMR spectra. a,** Sample Ru/TiO_2-8 with 10 kHz spinning. **b-e,** Deconvoluted signals of Ti-OD groups (**b**), Ru-D_{atop} (**c**), weakly bonded D to Ru (**d**) and total simulated spectra (**e**). **f,** Sample Ru/TiO_2-8 with 4 kHz spinning. **g-k,** Deconvoluted signals of Ti-OD groups (**g**), Ru-D_{atop} (**h**), weakly bonded D to Ru (**i**) and total simulated spectra (**k**).

Comparison of deconvolution results at different MAS rates (Supplementary Figure 14) shows reproducibility.

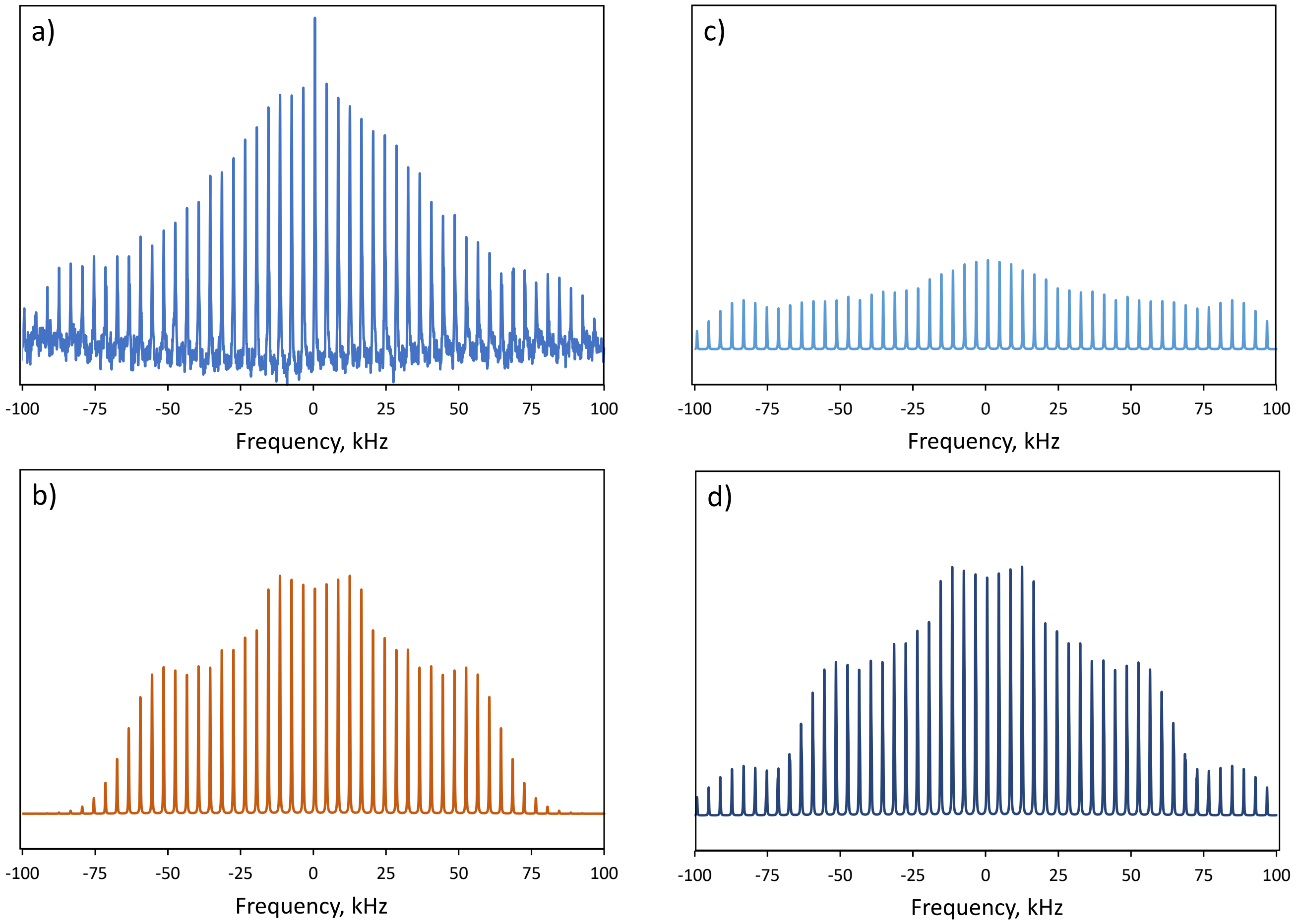

Supplementary Figure 15. **^{2}H MAS NMR spectra of TiO_2 support at 4 kHz spinning rate**. **a**, experimental spectra; **b-d**, deconvolution results: component 1 (δ_{iso}=6.8ppm) (b); component 2 (δ_{iso}=10.1ppm) (**c**) and simulated spectrum (**d**). Details in Supplementary Table 2.

Pure TiO_2 was deuterated with D_2O initially to create a sufficient number of Ti-OD groups and only then reduced in D_2 at 300 °C. Experimental spectra (Supplementary Figure 15) show that the resulting spectra are a superposition of at least two signals with different quadrupole coupling constants (Supplementary Table 2). Previous ^{1}H MAS NMR data[15] showed that anatase TiO_2 has Ti-OH signals at 1.9 ppm (external surface) and at 6.8 and 11.5 ppm. The last two signals correspond to the OH group in internal defects that are perturbed by interactions with neighboring lattice oxygen: Ti-OH…O_{lat}. In our case, spectra refinement identifies two major peaks at 6.8 and 10.1 ppm (Supplementary Table 2). The first has a higher intensity consistent with previous data.[15] The peak at 6.8 ppm has smaller Q_{cc} and η values compared to the second component at 10.1 ppm (Q_{cc} of 99.4 vs 141.4 kHz). A further downfield shifted peak (10.1 ppm) represents Ti-OD groups embedded in a more polar and asymmetric environment,

leading to these high values of Q_{cc} and η, compared to the 6.8 ppm peak. The reason for that may be hydrogen bonding or the proximity of a Ti-O⁻ group. Evidently, both Ti-OD groups do not produce a signal with low Q_{cc} value of ~70 kHz and very symmetrical EFG (η~0.1), which is typical for Ru-D_{atop} species.

Supplementary Table 2. **^{2}H MAS NMR data and EFG parameters for pure TiO_2 support (spinning rate 4 kHz).**

Parameter	peak 1	peak 2
δ_{iso}, ppm	6.8	10.1
relative intensity, a.u.	1	0.43
δ_{iso}, kHz	0.52	0.77
Q_{cc}, kHz	99.4	141.4
η	0.72	0.85
V_{xx}, 10^{20} V/m^2	-1.99	-1.58
V_{yy}, 10^{20} V/m^2	-12.37	-18.87
V_{zz}, 10^{20} V/m^2	14.37	20.45

Supplementary Table 3. **^{2}H MAS NMR data and EFG parameters for Ru/TiO_2 -8 sample at two different spinning rates.**

Parameter	4 kHz MAS			10 kHz MAS		
	Ti-OD	Ru-D_{atop}	“weak” D	Ti-OD	Ru-D_{atop}	“weak” D
δ_{iso}, ppm	6.75	2.22	-59.14	6.88	2.32	-58.15
Relative intensity, a.u.	1	0.68	0.29	1	0.76	0.18
δ_{iso}, kHz	0.52	0.17	-4.53	0.53	0.18	-4.48
Q_{cc}, kHz	119.4	71.4	0	124.9	73.48	0
η	0.99	0.11	0	0.99	0.19	0
V_{xx}, 10^{20} V/m^2	0.086	-4.59	-	-0.09	-4.30	-
V_{yy}, 10^{20} V/m^2	-17.18	-5.73	-	-17.97	-6.32	-
V_{zz}, 10^{20} V/m^2	17.27	10.32	-	18.06	10.63	-

NMR spectra measured at 4 kHz MAS have a more complex baseline, compare to 10 kHz (Supplementary Figure 14). Thus, the quadrupole and EFG parameters estimated at 10 kHz should be

less affected by baseline variation and should be more reliable to compare with DFT (Supplementary Table 4). Nonetheless, the parameters at both MAS rates were consistent with only small deviations.

Supplementary Table 4. **EFG parameters for Ru-D complex measured experimentally by ^{2}H MAS NMR and estimated using DFT.**

Parameter	^{2}H MAS NMR (Ru/TiO_2-8)*	DFT Ru_{12}/TiO_2	DFT Ru_{12}	DFT Ru_6
δ_{iso}, ppm	2.32	-	-	-
Q_{cc}, kHz	73.5	79.1	68.0	67.9
η	0.19	0.12	0.05	0.02
V_{xx}, 10^{20} V/m^2	-4.30	-6.37	-5.17	-5.02
V_{yy}, 10^{20} V/m^2	-6.32	-5.06	-4.65	-4.80
V_{zz}, 10^{20} V/m^2	10.63	11.43	9.83	9.82

* - Quadrupole parameters shown for Ru/TiO2-8 samples at 10 kHz MAS rate (see Supplementary Table 3).

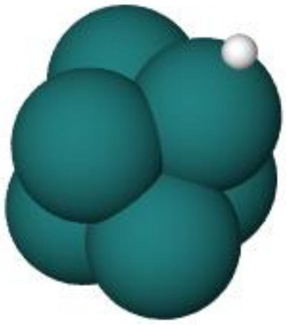

(a)

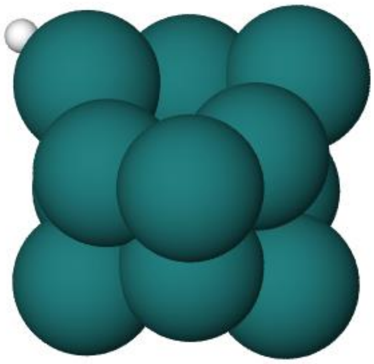

(b)

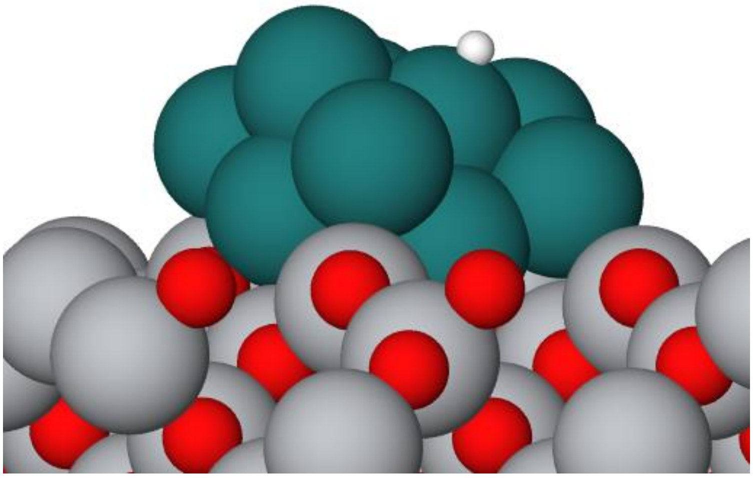

(c)

Supplementary Figure 16. **Structures for estimating the EFG of ^{2}H on different Ru clusters**. (a) ^{2}H on an isolated Ru_6. (b) ^{2}H on an isolated Ru_{12}. (c) ^{2}H on supported $Ru_{12}/TiO_2(101)$.

Small Ru clusters have much more symmetrical EFG (η~0.02), compared to experimental results. A larger Ru_{12} cluster has slightly more asymmetry and correlates reasonably with experiments. The addition of the support to the model (Supplementary Figure 16c) increases V_{zz} due to the electrostatic influence of O^{2-} in the second coordination sphere of Ru. This leads to a small disagreement between the model and experiment in Q_{cc} and V_{zz} values. The Ru surface has multiple Ru-D fragments on neighboring surface atoms leading to reduced effective V_{zz} value. Also, more distant Ru atoms from the support in the third and fourth layers show smaller V_{zz}, closer to a free-standing cluster. Thus, the small variation in particle morphology from a sphere to a flat monolayer could strongly deviate the experimental V_{zz} from model predictions.

Effect of sample evacuation on ^{2}H MAS NMR spectra

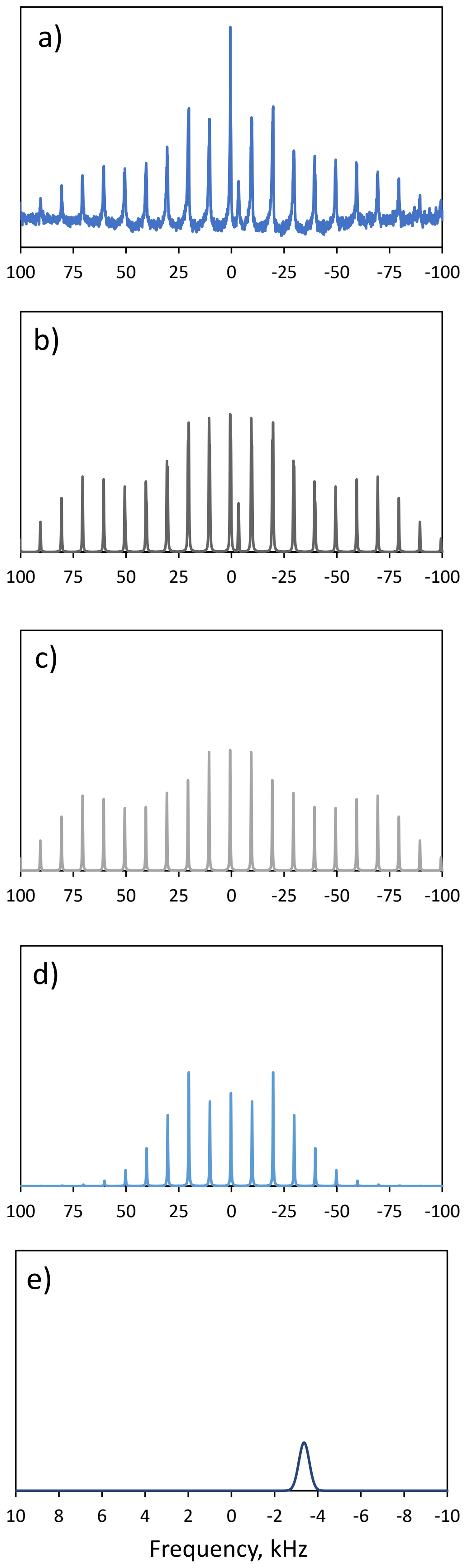

Supplementary Figure 17. **^{2}H MAS NMR spectra of Ru/TiO_2-12 with 10 kHz spinning after evacuation at 100 °C.** (a) and total fitted spectra (b); deconvoluted signal of Ti-OD groups (c), Ru-D_{atop} (d), weakly bonded D to Ru (e).

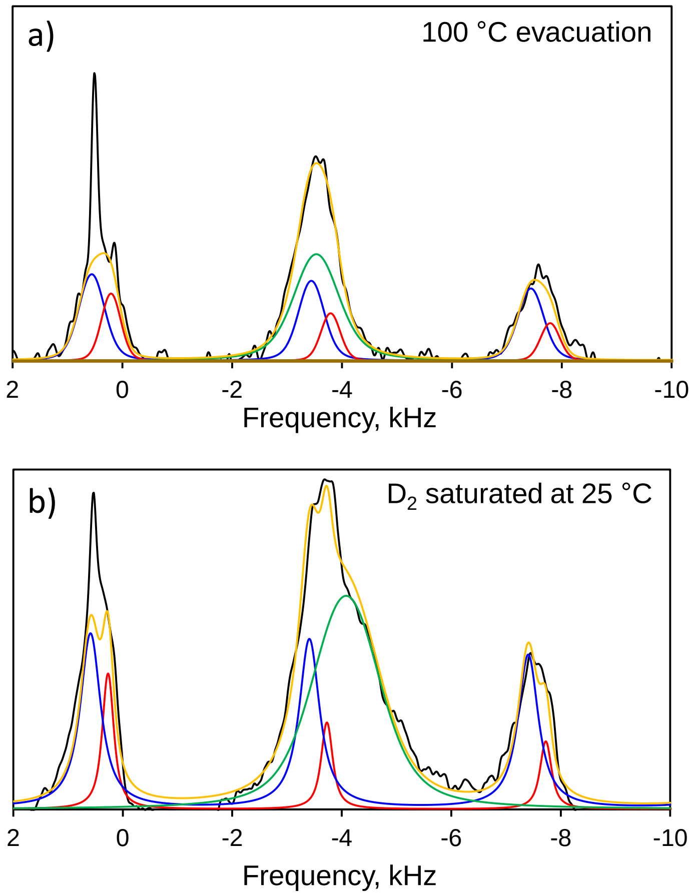

Supplementary Figure 18. **Effect of evacuation of ^{2}H MAS NMR spectra**. **a,b** ^{2}H NMR spectra of Ru/TiO_2-12 sample prereduced at 300 °C after evacuation at 100 °C (**a**) and after saturation with D_2 at 25 °C (**b**). 4 kHz MAS rate. Legend: black line – raw data, yellow – fitted spectra; blue – Ti-OD groups; red – Ru-D_{atop}; green – weakly bonded D.

After the evacuation at 100 °C, the intensity of all signals decreases slightly due to partial desorption of D_2. The relative ratio of Ti-OD and Ru-D_{atop} peaks remained unchanged, and they also show very similar δ_{iso} at 0.54 and 0.20 kHz (7.00 and 2.58 ppm), respectively. At the same time, the broad peak shifts from -4.10 kHz (-53.2 ppm) to -3.53 kHz (-45.8 ppm) and becomes narrower. This peak is therefore assigned to weakly bonded deuterium. It may be trapped on Ru surface sites with the participation of Ru conduction electrons or from Ti^{3+} sits on the partially reduced support.

Supplementary Table 5. **Comparison of signals relative intensity for Ru/TiO_2-12 sample after evacuation at 100 °C (MAS rate 4 kHz)**.

Treatment	Intensity distribution, a.u.		
	Ti-OD	Ru-D_{atop}	weakly bonded D
saturated with D_2 at 25 °C	0.62	0.26	0.11
evacuated 100 °C	0.65	0.28	0.065

Sample comparison

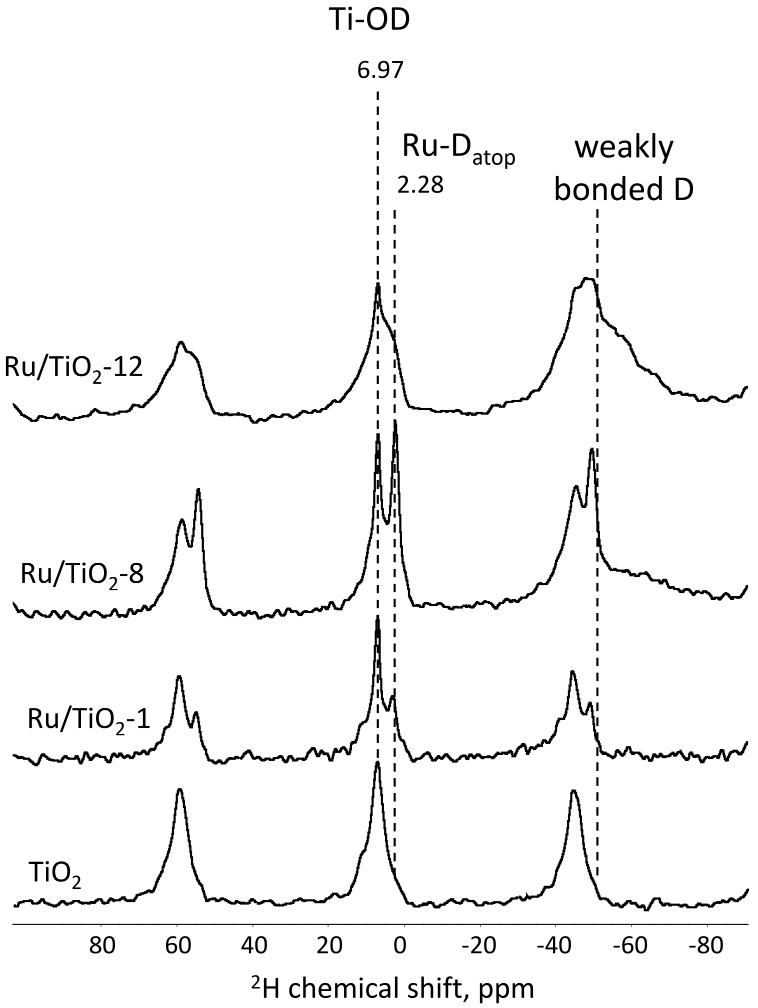

Supplementary Figure 19. **Comparison of ^{2}H MAS NMR spectra of different Ru/TiO_2 samples and TiO_2 support**. MAS rate 4 kHz.

Supplementary Table 6. **Quantitative analysis of ^{2}H MAS NMR spectra of different Ru/TiO_2 samples and TiO_2 support.**

**Sample**	MAS rate, kHz	**Intensity distribution, a.u.**		
		Ti-OD	Ru-D$_{atop}$	weakly bonded Ru
TiO_2	4	1	0	0
Ru/TiO_2-1	4	0.78	0.22	~0.00
	10	0.8	0.18	0.02
Ru/TiO_2-8	4	0.51	0.35	0.15
	10	0.52	0.39	0.09
Ru/TiO_2-12	4	0.62	0.26	0.11

Supplementary Table 7. **Absolute intensities of ^{2}H MAS NMR spectra components measured at 10 kHz MAS rate.**

**Sample**	**Absolute intensity, 10^{12} kHz**		
	Ti-OD	Ru-D_{atop}	weakly bonded Ru
Ru/TiO_2-1	1.85	0.43	0.03
Ru/TiO_2-8	1.06	0.80	0.18

Absolute intensities of Ru-D_{atop} and "weakly" bonded deuterium are higher for Ru/TiO_2-8, compared to Ru/TiO_2-1. Interestingly, NMR also reflects a smaller density of Ti-OD groups on Ru/TiO_2-8, consistent with DRIFTS results (Supplementary Figure 10).

FTIR spectra

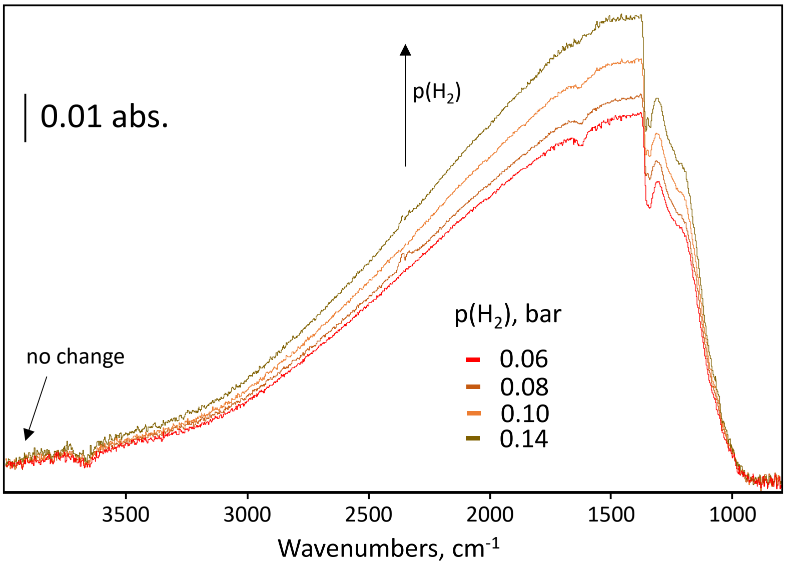

Supplementary Figure 20. **FTIR spectra of Ru/TiO_2-1 in H_2/He flow at different H_2 partial pressures at 250 °C**. The spectra in pure He at 250 ° C were used for subtraction.

Spectra taken at different partial pressures of H_2 at 250 °C show that the electrons concentration in shallow traps (ST) increases with p(H_2). Interestingly, no differences were found at high frequency due to the absence of conduction band (CB) electrons at these pressures.

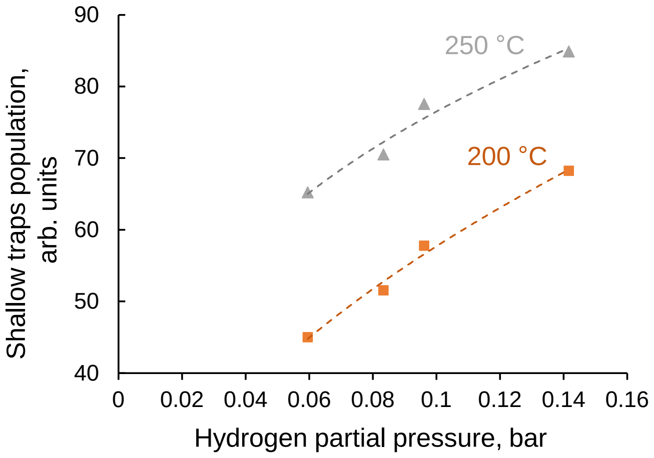

Supplementary Figure 21. **Shallow trap population estimated from FTIR spectra vs. hydrogen partial pressure at 200 and 250 °C.**

Quantitative analysis of the ST peak (Supplementary Figure 21) shows that increasing the temperature and hydrogen pressure leads to more electrons transferred to ST from H_2. At 200 °C, this dependence has a square root form $\sim p(H_2)^{0.5}$, while at 250 °C, it tends to saturate, $\sim p(H_2)^{0.3}$.

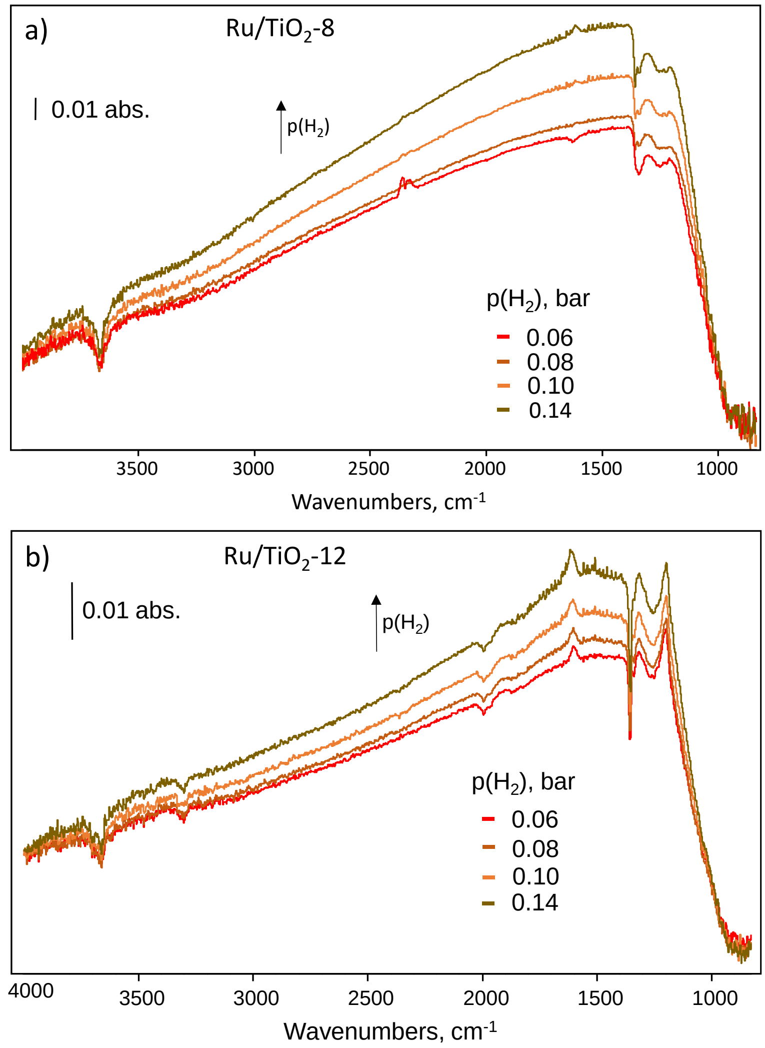

Supplementary Figure 22. **FTIR spectra of hydrogen spillover**. **a,b** Ru/TiO_2-8 (**a**) and Ru/TiO_2-12 (**b**) samples in H_2/He flow at different H_2 partial pressures at 250 °C. Spectra in pure He at 250 °C were used for subtraction.

Both NH_3-treated samples (Supplementary Figure 22) show a much broader band with non-zero intensity at high wavenumbers (~4000-3800 cm^{-1}). That is because delocalized CB electrons form via hydrogen spillover in addition to ST filling. Also, both samples reveal a small peak at 1602-1604 cm^{-1} due to δ(HOH) deformational vibration in water formed by protonation of the surface Ti-OH groups. This is also accompanied by a decrease in the 3661 cm^{-1} peak of ν(OH) vibration of these groups. Based on this observation, we assume that extensive support reduction is happening over Ru/TiO_2-8 and Ru/TiO_2-12, while for Ru/TiO_2-1, this process is retarded and does not lead to water.

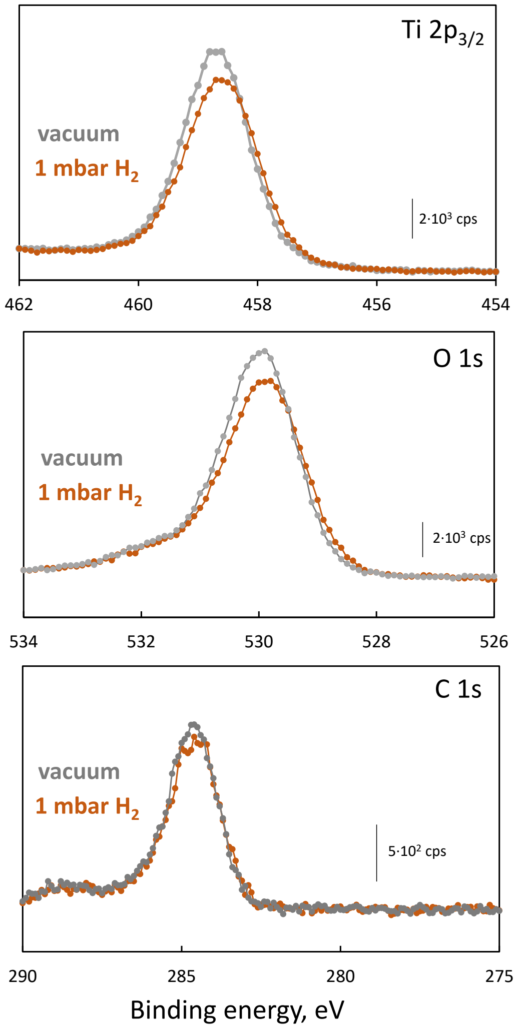

Supplementary Figure 23. **NAP-XPS of pure TiO_2 support**.

The spectra for pure TiO_2 support (Supplementary Figure 23) show the standard signal for anatase at 458.69 eV for Ti 2$p_{3/2}$ (Ti^{4+} cations) and 530.0 eV peak for O 1*s* (O^{2-} anions in the lattice).[16] The addition of 1 mbar of H_2 at 200 °C leads to a shift in binding energies to lower values by 0.08 - 0.1 eV due to hydrogen binding. Oxygen vacancies on the TiO_2 surface and subsurface can chemisorb hydrogen,[17] leading to the observed small peak shift.

Supplementary Table 8. **NAP-XPS spectra analysis.**

Sample	Conditions		Ru		Ti		O 1*s*	
	T, °C	$p(H_2)$, mbar	$3d_{5/2}$	$3d_{3/2}$	$2p_{3/2}$	$2p_{1/2}$	peak 1	peak 2
Ru/TiO_2-1	25	0*	279.72	284.00	458.64	464.24	529.91	531.71
	200	0	279.70	284.58	458.55	464.1	529.83	531.65
	200	1	279.82	283.92	458.56	464.11	529.85	531.62
Ru/TiO_2-8	25	0	280.16	284.66	458.83	464.41	530.5	532.23
	200	0	280.12	284.62	458.69	464.26	529.99	531.7
	200	1	280.73	285.13	458.54	464.12	529.84	531.58
TiO_2	200	0	-	-	458.69	464.33	529.99	531.8
	200	1	-	-	458.61	464.27	529.89	531.73

* - spectra were measured in UHV.

Quantitative analysis (Supplementary Table 8) shows that the hydrogen-induced shift in Ti $2p_{3/2}$ and O 1*s* peaks position for Ru/TiO_2-1 is below the detection level ~0.01-0.02 eV. On the more reducible Ru/TiO_2-8, the shift is 0.15 eV. Similar sensitivity to H_2 was observed in the Ru 3*d* region. On the Ru/TiO_2-1, the Ru $3d_{5/2}$ peak shifts to higher binding energies by 0.12 eV. On the Ru/TiO_2-8, the shift is 0.61 eV.

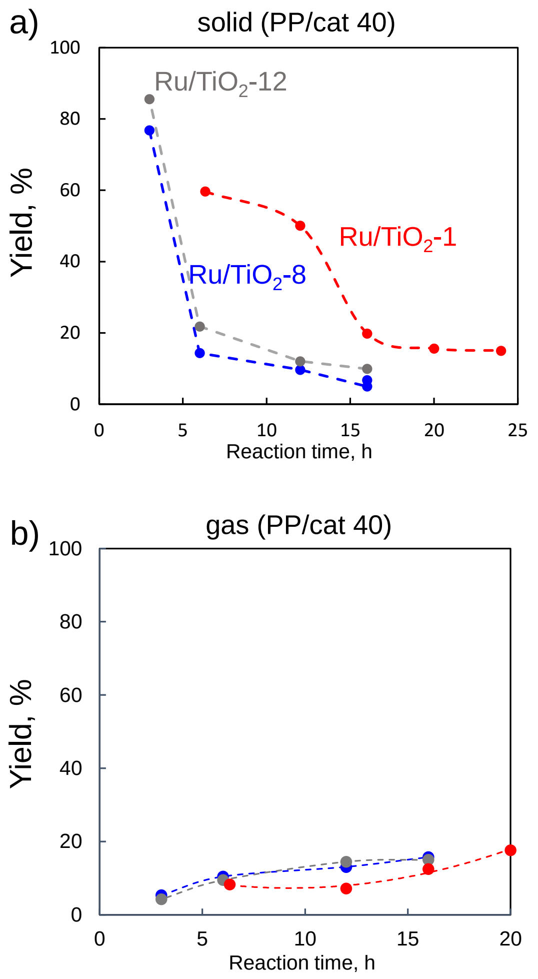

Supplementary Figure 24. **PP hydrogenolysis kinetics at PP/catalyst ratio 40**. **a,b** solid residue (**a**) and gas (**b**) yields for PP conversion over different Ru/TiO_2 samples.
Conditions: 250 °C, 30 bar H_2, 2 g PP, 50 mg catalyst.

At low catalyst loading (a PP/catalyst ratio 40), a small fraction of gas is produced. The initial solid consumption is much faster over Ru/TiO_2-12 and Ru/TiO_2-8 in line with the higher liquid yields over these samples.

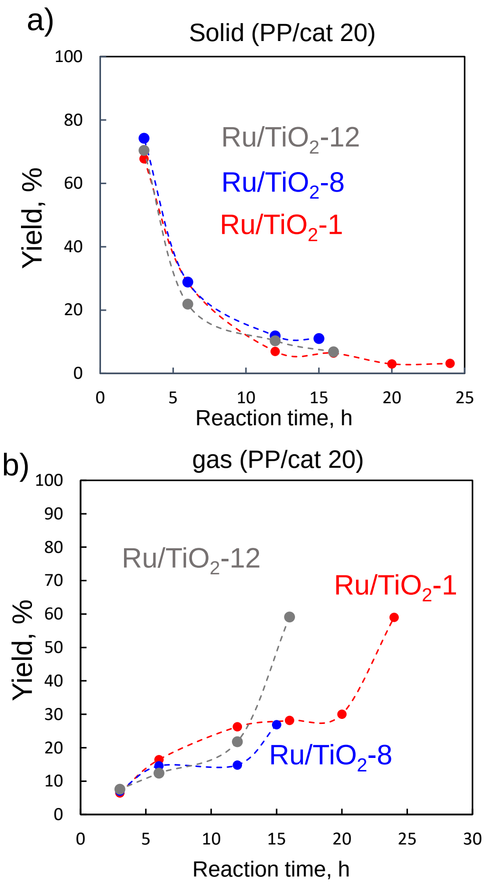

Supplementary Figure 25. **PP hydrogenolysis kinetics at PP/catalyst ratio 20. a,b** Solid residue (**a**) and gas (**b**) yields for PP conversion over different Ru/TiO_2 samples at higher catalyst loading. Conditions: 250 °C, 30 bar H_2, 2 g PP, 100 mg catalyst.

At higher catalyst loading (a PP/catalyst ratio 20), the solid residue consumption is comparable for all samples. The system reaches maximum activity, and the reaction rate is limited by mass transfer. The same pattern is seen for the liquid yield vs. reaction time. The gas yield increases slowly for all samples until a point where the liquid M_w is low that the liquid to gas conversion starts.

Supplementary Table 9. **Molecular weight distribution of liquid products formed over various Ru/TiO_2 catalysts at different reaction times at a constant PP/catalyst ratio of 20**.

Catalyst	Reaction time, h	M_w, 10^{-3} g/mol	M_n, 10^{-3} g/mol	PDI
Ru/TiO_2-1	3	14.00	5.26	2.7
	6	2.67	1.17	2.3
	12	0.97	0.695	1.4
	16	0.90	0.68	1.3
Ru/TiO_2-8	3	20.58	6.58	-
	6	10.92	3.64	4.4
	12	0.86	0.62	1.2
	16	0.88	0.65	1.2
Ru/TiO_2-12	3	13.22	4.33	3.1
	6	15.62	4.67	3.3
	12	1.81	1.03	1.7
	16	0.91	0.67	1.4

Reaction conditions: 250 °C, 30 bar H_2, 2 g PP, 100 mg catalyst.

Supplementary Table 10. **Molecular weight distribution of liquid products formed over various Ru/TiO_2 catalysts at different reaction times at a constant PP/catalyst ratio of 40**.

Catalyst	Reaction time, h	M_w, 10^{-3} g/mol	M_n, 10^{-3} g/mol	PDI
Ru/TiO_2-1	6	14.34	4.68	3.1
	12	18.70	5.46	3.4
	16	8.97	3.21	2.8
	20	2.04	1.49	1.4
	24	2.38	1.22	2.0
Ru/TiO_2-8	3	13.65	4.38	3.1
	6	9.91	3.06	3.2
	12	6.18	2.65	2.3
	16	7.10	1.78	4.0
Ru/TiO_2-12	3	25.25	9.44	2.7
	6	2.75	1.49	1.8
	12	3.02	1.49	2.0
	16	4.11	1.93	2.1

Reaction conditions: 250 °C, 30 bar H_2, 2 g PP, 50 mg catalyst.

Supplementary Table 11. **Yields of C_1-C_6 gas product**.

	methane	ethane	propane	butane and isobutane	pentanes	hexanes
Ru/TiO_2-1						
3h	4.7	0.6	0.2	0.2	0	0
6h	11.6	1.4	0.6	0.5	0.3	0.3
12h	18.3	2.5	1.0	0.9	0.5	0.3
16h	19.2	2.7	1.2	1.1	0.6	0.4
Ru/TiO_2-8						
3h	4.7	0.7	0.3	0.3	0.1	0.05
6h	10.3	1.3	0.6	0.5	0.2	0.09
12h	18.0	2.4	1.0	0.9	0.5	0.4
15h	18.6	2.5	1.1	0.9	0.5	0.4
Ru/TiO_2-8						
3h	5.3	0.7	0.3	0.3	0.2	0.05
6h	8.7	1.2	0.5	0.4	0.1	0.04
12h	15.5	2.0	0.8	0.6	0.3	0.08
16h	41.8	8.5	0.5	1.1	0.3	0.2

Reaction conditions: 250 °C, 30 bar H_2, 2 g PP, 100 mg catalyst.

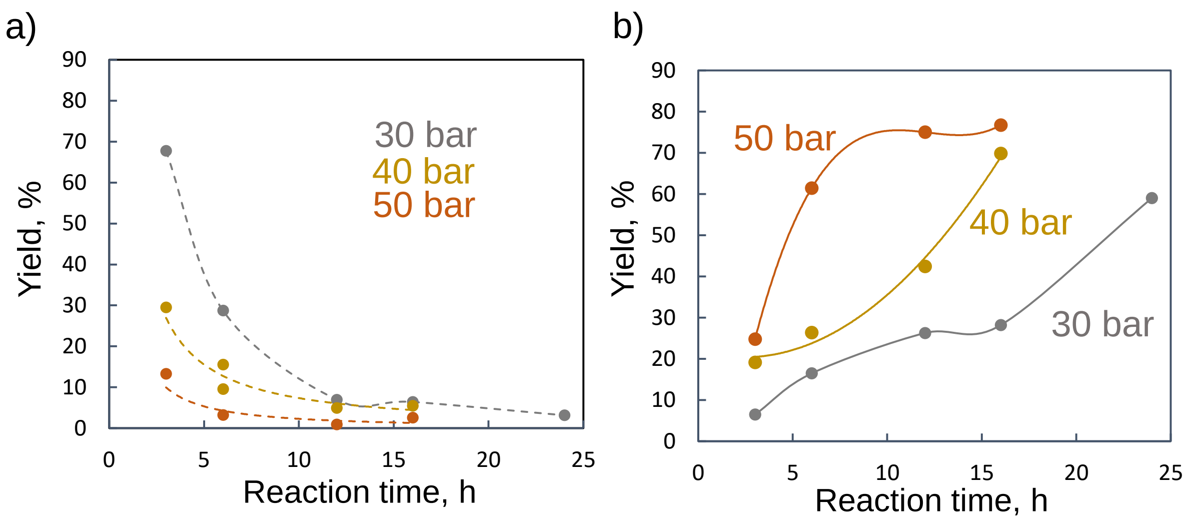

Supplementary Figure 26. **Product yield vs. time. a,** Solid residue. **b,** gas. PP conversion over Ru/TiO_2-1 at different hydrogen pressures at 250 °C, 2 g PP, and 100 mg catalyst.

Supplementary Table 12. **Molecular weight distribution of liquid products formed over Ru/TiO_2-1 catalyst at different reaction times and hydrogen pressures**.

$P(H_2)$, bar	Reaction time, h	M_w, 10^{-3} g/mol	M_n, 10^{-3} g/mol	PDI
30	6	14.34	4.68	3.1
	12	18.70	5.46	3.4
	16	8.97	3.21	2.8
	20	2.04	1.49	1.4
	24	2.38	1.22	2.0
40	3	12.26	3.97	3.1
	6	1.08	0.76	1.4
	12	0.83	0.65	1.3
	16	0.90	0.72	1.2
50	3	2.63	1.46	1.8
	6	0.78	0.62	1.3
	12	0.93	0.75	1.2
	16	0.91	0.73	1.2

Reaction conditions: 250 °C, 2 g PP, 100 mg catalyst.

Supplementary discussion I: kinetic model

Kinetic modeling can be useful to obtain a qualitative correlation of the hydrogen coverage, reaction rate, and H/D exchange. Following previous work,[18] we assume the following key steps in PP hydrogenolysis. Step 1 involves the PP dissociative chemisorption over a Ru-H group with the synchronous desorption of H_2 (rate constants k_1 and k_{-1}):

$$C_nH_{2n+2} + H_{ads} \leftrightarrow C_nH_{2n+2-1} + H_2 \quad (1),$$

where H_{ads} corresponds to an adsorbed hydrogen on the Ru-H group. Step 2 is the C-C bond breaking in the adsorbed intermediate (rate constant k_2):

$$C_nH_{2n+2-1} \rightarrow products \quad (2).$$

Using the quasi-stationary approximation for C_nH_{2n+2-1}, we get:

$$[C_nH_{2n+2-1}] = \frac{k_1k_2[PP][H_{ads}]}{k_{-1}[H_2] + k_2} \quad (3),$$

where [PP] is the concentration of polymer, $[H_{ads}]$ is the concentration of adsorbed hydrogen, and $[H_2]$ is concentration of H_2. In a narrow pressure range, the Temkin isotherm[19] describes $[H_{ads}]$ as a function of p_H:

$$[H_{ads}] = \frac{1}{f} ln(a_0p_H) \quad (4),$$

where f and a_0 are constants. In this approximation a_0p_H is always above 1. The reaction rate is $r = k_2[C_nH_{2n+2-1}]$ and thus:

$$r = \frac{k_1k_2[PP]\frac{1}{f}ln(a_0p_H)}{k_{-1}p_H + k_2} \quad (5).$$

At low p_H, r depends linearly on $\ln(p_H)$, while at high p_H the rate should be zero. As a result, the experimental r vs. p_H curve should have a maximum consistent with experiments on light alkanes.[20]

Using the rate expression, we can estimate the kinetic isotope effect (KIE). Breaking of Ru-H or Ru-D bond in step 1 during the polymer binding can be analyzed using transition state theory:

$$k_1^H = \frac{kT}{h}e^{\frac{-\Delta G_H^{\neq}}{RT}} = \frac{kT}{h}\frac{Q^{\neq}}{Q_{polymer}Q_{Ru-H}}e^{\frac{-\Delta E_H^0}{RT}} \quad (6),$$

where k_1^H is the rate constant in H_2, ΔG_H^0 is the Gibbs energy of activation, Q stands for the partition functions of the transition state, the initial polymer, and the surface hydrogen; ΔE_H^0 is the difference in zero-point energy between the reactants and the transition state. Assuming that the transition state and polymer in presence of deuterium have the same partition functions, the difference arises from switching between Ru-H and Ru-D. We can simplify as follows:

$$\frac{k_1^H}{k_1^D} = \frac{1 - e^{\frac{-h\nu_{Ru-H}}{kT}}}{1 - e^{\frac{-h\nu_{Ru-D}}{kT}}} e^{\frac{h}{2kT}(\nu_{Ru-H} - \nu_{Ru-D})} \quad (7),$$

where k_1^D is the rate constant in D_2; ν_{Ru-H} and ν_{Ru-D} are vibrational frequencies of Ru-H and Ru-D vibrations. Using the harmonic approximation:

$$\frac{\nu_{Ru-H}}{\nu_{Ru-D}} \cong \sqrt{\frac{m_H}{m_D}}\sqrt{\frac{m_{Ru}+m_H}{m_{Ru}+m_D}} = 0.7 \quad (8).$$

With a standard ν_{Ru-H} value of ~2000 cm^{-1}, the KIE will reach 2.3 at 250 °C. At our experimental conditions, the polymer is deuterated and possesses C-D bonds due to quasi-equilibrated isotope exchange with gaseous D_2. This will further increase the isotope effect of the forward step 1, since the C-D bond is harder to break. The reverse step 1 rate constants ratio (k_{-1}^H and k_{-1}^D) can be estimated using a similar procedure using the frequencies of gaseous H_2 and D_2 leading to:

$$\frac{k_{-1}^H}{k_{-1}^D} = \frac{1-e^{\frac{-h\nu_{H-H}}{kT}}}{1-e^{\frac{-h\nu_{D-D}}{kT}}} e^{\frac{h}{2kT}(\nu_{H-H}-\nu_{D-D})} = 5.81 \ \ (9).$$

The C-C bond breaking in the adsorbed polymer should not be influenced directly by the H_2/D_2 exchange and

$$\frac{k_2^H}{k_2^D} \cong 1 \quad (10).$$

Qualitative modeling results in Supplementary Figure 27 show that a higher hydrogen pressure will cause: i) a higher reaction rate and ii) a lower KIE. It seems that the sufficient condition for KIE to decline with p_H is the existence of some isotope effect on the reverse step 1, which is here associated with breaking the hydrogen molecule. If KIE decreases, D_2 has a smaller impact on the net rate leading to similar molecular weight distributions of liquid products.

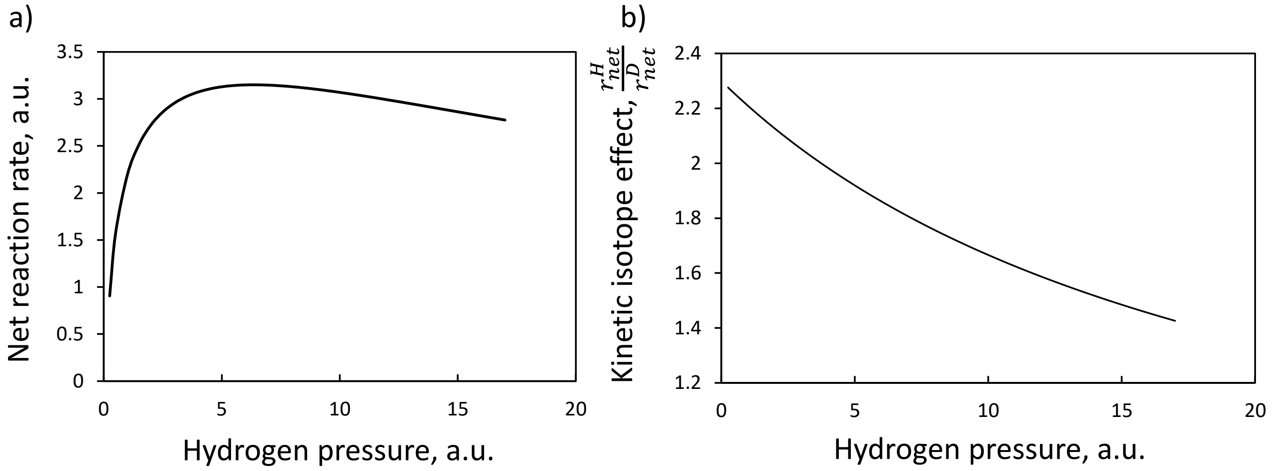

Supplementary Figure 27. **Modeling of hydrogenolysis kinetics at different hydrogen coverage**. **a,** dependence of net reaction rate; **b,** ratio r_{net}^H to r_{net}^D as a function of hydrogen coverage. Parameters: $k_1 = 1\ M^{-1}s^{-1}, k_{-1} = 1\ M^{-1}s^{-1}, C_{polymer} = 1\ M, k_2 = 20\ s^{-1}, a_0 = 10\ bar^{-1},\ f = 1.$

Our experimental results on PP hydrogenolysis corresponds to a positive effect of p_H with no inhibition. This is a stark difference between polymers and small alkanes, where H_2 usually serves as an inhibitor.[21] Because of the weak alkane binding to the metal surface, θ_H is usually close to saturation at hydrogen pressure above 10 bar. For PP, the binding is stronger, leading to small θ_H and a beneficial effect of H_2 pressure on the net rate. The Ru/TiO_2-8 catalyst has a higher surface hydrogen coverage based on the NMR data. Thus, it should have a lower KIE and be less sensitive to H_2/D_2 substitution. It is indeed evident from the molecular weight distributions of the liquid products. Over Ru/TiO_2-1 with a lower θ_H, the KIE is expected to be more pronounced. This is manifested with a shift in the M_w to higher values and a heavier liquid.

Supplementary Table 13. **Comparison with previously published results.**

Catalyst	PP/catalyst weight ratio, g/g	M_n of PP, kDa	T, °C	t, h	Yield of C_1-C_4 gas, %	Yield of liquid, %	Reference
Ru/CeO_2	17	~5	240	72	17	83	[22]
Ru/C	14	~97	250	8	42.9	35.7	[23]
Ru-TiO_2-1	20	~67	250	12	26.3	65.4	[24]
Ru-TiO_2-1	40	~67	250	20	17.6	63.1	[24]
Ru-TiO_2-8	20	~67	250	12	14.8	63.2	this work
Ru-TiO_2-8	40	~67	250	6	9.5	74.1	this work

Supplementary references

1 Frenkel, A. I., Yevick, A., Cooper, C. & Vasic, R. Modeling the structure and composition of nanoparticles by extended X-ray absorption fine-structure spectroscopy. *Annu. Rev. Anal. Chem.* **4**, 23-39 (2011).

2 Karim, A. M. *et al.* Correlating particle size and shape of supported Ru/γ-Al_2O_3 catalysts with NH_3 decomposition activity. *J. Am. Chem. Soc.* **131**, 12230-12239 (2009).

3 Fàbrega, C. *et al.* Tuning the fermi level and the kinetics of surface states of TiO2 nanorods by means of ammonia treatments. *J. Phys. Chem. C* **117**, 20517-20524 (2013).

4 Takeuchi, M., Tsukamoto, T., Kondo, A. & Matsuoka, M. Investigation of NH_3 and NH_4^+ adsorbed on ZSM-5 zeolites by near and middle infrared spectroscopy. *Catal. Sci. Technol.* **5**, 4587-4593 (2015).

5 Hadjiivanov, K. FTIR study of CO and NH_3 co-adsorption on TiO_2 (rutile). *Appl. Surf. Sci.* **135**, 331-338 (1998).

6 Jones, C. M., Johnson, C. R., Asher, S. A. & Shepherd, R. E. Resonance Raman studies of the excited electronic states of $(CN)_5Fe^{III}(imidazole)_2$-and $(NH_3)_5Ru^{III}(imidazole)^{3+}$. *J. Am. Chem. Soc.* **107**, 3772-3780 (1985).

7 Amores, J. G., Escribano, V. S., Ramis, G. & Busca, G. An FT-IR study of ammonia adsorption and oxidation over anatase-supported metal oxides. *Appl. Catal. B: Environ.* **13**, 45-58 (1997).

8 Hadjiivanov, K., Saur, O., Lamotte, J. & Lavalley, J.-C. FT-IR spectroscopic study of NH_3 and CO adsorption and coadsorption on TiO_2 (anatase). *Z. Phys. Chem.* **187**, 281-300 (1994).
9 Mazzolini, P. *et al.* Vibrational–electrical properties relationship in donor-doped TiO_2 by Raman spectroscopy. *J. Phys. Chem. C* **120**, 18878-18886 (2016).
10 Hadjiivanov, K. *et al.* FTIR Study of CO Interaction with Ru/TiO_2 Catalysts. *J. Catal.* **176**, 415-425 (1998).
11 Elmasides, C., Kondarides, D., Grünert, W. & Verykios, X. XPS and FTIR study of Ru/Al_2O_3 and Ru/TiO_2 catalysts: reduction characteristics and interaction with a methane− oxygen mixture. *J. Phys. Chem. B* **103**, 5227-5239 (1999).
12 Guglielminotti, E. & Bond, G. C. Effect of oxidation–reduction treatments on the infrared spectra of carbon monoxide chemisorbed on a Ru/TiO_2 catalyst. *J. Chem. Soc., Faraday Trans.* **86**, 979-987 (1990).
13 Du, P. *et al.* The effect of surface OH-population on the photocatalytic activity of rare earth-doped P25-TiO_2 in methylene blue degradation. *J. Catal.* **260**, 75-80 (2008).
14 Vuk, A. Š., Ješe, R., Gaberšček, M., Orel, B. & Dražič, G. Structural and spectroelectrochemical (UV–vis and IR) studies of nanocrystalline sol–gel derived TiO_2 films. *Sol. Energy Mater. Sol. Cells* **90**, 452-468 (2006).
15 Sun, X. *et al.* Solid-state NMR investigation of the $^{16/17}O$ isotope exchange of oxygen species in pure-anatase and mixed-phase TiO_2. *Chem. Phys. Lett.* **594**, 34-40 (2014).
16 Abdel-Mageed, A. M. *et al.* Steering the selectivity in CO_2 reduction on highly active Ru/TiO_2 catalysts: Support particle size effects. *J. Catal.* **401**, 160-173 (2021).
17 Di Valentin, C., Pacchioni, G. & Selloni, A. Reduced and n-type doped TiO_2: nature of Ti^{3+} species. *J. Phys. Chem. C* **113**, 20543-20552 (2009).
18 Shang, S. & Kenney, C. Steady-state and transient kinetic studies of ethane hydrogenolysis over Ru/Al_2O_3. *J. Catal.* **134**, 134-150 (1992).
19 Chu, K. H. Revisiting the Temkin Isotherm: Dimensional Inconsistency and Approximate Forms. *Ind. Eng. Chem. Res.* **60**, 13140-13147 (2021).
20 Shi, H., Gutierrez, O. Y., Zheng, A., Haller, G. L. & Lercher, J. A. Mechanistic Pathways for Methylcyclohexane Hydrogenolysis over Supported Ir Catalysts. *J. Phys. Chem. C* **118**, 20948-20958 (2014).
21 Flaherty, D. W. & Iglesia, E. Transition-state enthalpy and entropy effects on reactivity and selectivity in hydrogenolysis of n-alkanes. *J. Am. Chem. Soc.* **135**, 18586-18599 (2013).
22 Nakaji, Y. *et al.* Low-temperature catalytic upgrading of waste polyolefinic plastics into liquid fuels and waxes. *Appl. Catal. B: Environ.* **285**, 119805 (2021).
23 Rorrer, J. E., Troyano-Valls, C., Beckham, G. T. & Román-Leshkov, Y. Hydrogenolysis of Polypropylene and Mixed Polyolefin Plastic Waste over Ru/C to Produce Liquid Alkanes. *ACS Sustain. Chem. Eng.* **9**, 11661-11666 (2021).
24 Kots, P. A. *et al.* Polypropylene Plastic Waste Conversion to Lubricants over Ru/TiO_2 Catalysts. *ACS Catal.* **11**, 8104-8115 (2021).
